# Computational Methods for Modeling Lipid-Mediated Active Pharmaceutical Ingredient Delivery

**DOI:** 10.1021/acs.molpharmaceut.4c00744

**Published:** 2025-01-29

**Authors:** Markéta Paloncýová, Mariana Valério, Ricardo Nascimento Dos Santos, Petra Kührová, Martin Šrejber, Petra Čechová, Dimitar A. Dobchev, Akshay Balsubramani, Pavel Banáš, Vikram Agarwal, Paulo C. T. Souza, Michal Otyepka

**Affiliations:** 1 Regional Center of Advanced Technologies and Materials, Czech Advanced Technology and Research Institute (CATRIN), Palacký University Olomouc, Šlechtitelů 27, 779 00 Olomouc, Czech Republic; 2 Laboratoire de Biologie et Modélisation de la Cellule, CNRS, UMR 5239, Inserm, U1293, Université Claude Bernard Lyon 1, Ecole Normale Supérieure de Lyon, 46 Allée d’Italie, 69364 Lyon, France; 3 Centre Blaise Pascal de Simulation et de Modélisation Numérique, Ecole Normale Supérieure de Lyon, 46 Allée d’Italie, 69364 Lyon, France; 4 mRNA Center of Excellence, Sanofi, 69280 Marcy-l'Étoile, France; 5 IT4Innovations, VŠB − Technical University of Ostrava, 17. listopadu 2172/15, 708 00 Ostrava-Poruba, Czech Republic; 6 mRNA Center of Excellence, Sanofi, Waltham, Massachusetts 02451, United States

**Keywords:** lipid nanoparticle, lipid nanocarrier, liposome, vesicle, ionizable lipid, molecular simulation

## Abstract

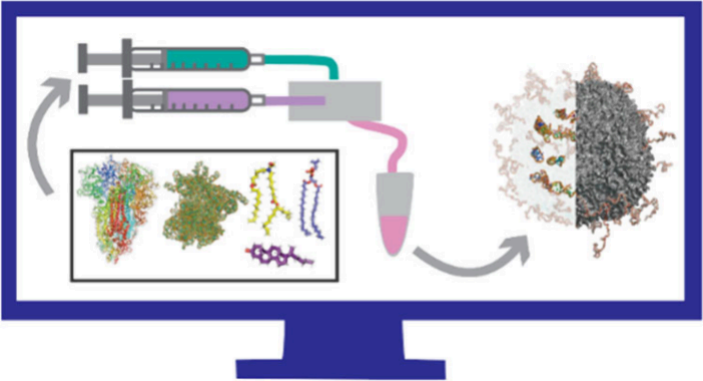

Lipid-mediated delivery of active pharmaceutical ingredients (API) opened new possibilities in advanced therapies. By encapsulating an API into a lipid nanocarrier (LNC), one can safely deliver APIs not soluble in water, those with otherwise strong adverse effects, or very fragile ones such as nucleic acids. However, for the rational design of LNCs, a detailed understanding of the composition–structure–function relationships is missing. This review presents currently available computational methods for LNC investigation, screening, and design. The state-of-the-art physics-based approaches are described, with the focus on molecular dynamics simulations in all-atom and coarse-grained resolution. Their strengths and weaknesses are discussed, highlighting the aspects necessary for obtaining reliable results in the simulations. Furthermore, a machine learning, i.e., data-based learning, approach to the design of lipid-mediated API delivery is introduced. The data produced by the experimental and theoretical approaches provide valuable insights. Processing these data can help optimize the design of LNCs for better performance. In the final section of this Review, state-of-the-art of computer simulations of LNCs are reviewed, specifically addressing the compatibility of experimental and computational insights.

## 1 Lipid-Mediated Delivery

The delivery of active pharmaceutical ingredients (APIs) faces multiple challenges, including permeation through multiple compartments and reaching the intended sites,^1^ overcoming low water solubility or susceptibility to instability in the *e.g.* gastrointestinal tract^2^ or low topical permeability. The targeted delivery can increase the treatment efficiency and reduce unwanted side effects in undesired locations.^3^ The process of delivery is governed by the physicochemical properties of the APIs, making it crucial to preserve their functionality. This task is particularly challenging for fragile macromolecules, such as proteins or nucleic acids such as messenger RNA (mRNA), which are highly promising APIs but prone to degradation.

The encapsulation of APIs into lipid-based nanocarriers has revolutionized the landscape of API delivery,^4^ marking a significant milestone with the introduction of the first FDA-approved nanodrug Doxil. This breakthrough greatly enhanced the safety and delivery efficiency of doxorubicin by prolonging its circulation time in the body and preferentially targeting tumors, thereby reducing cardiotoxicity.^3,5^ However, while liposomes have proven effective for many applications,^6−8^ they may not be ideal for delivering nucleic acids due to their high negative charge and lability.^9^ Decades of research on RNA delivery^10,11^ culminated during the COVID-19 pandemic,^12−15^ allowing efficient vaccination of billions using lipid nanoparticles (LNPs) carrying mRNA. LNPs shield mRNA from enzymatic degradation and significantly enhance its cellular uptake and expression.^16,17^ This versatile technology has opened new avenues for vaccination against infectious diseases, cancer treatment, and gene therapies for rare diseases.^18−21^

The rapid expansion of administrable APIs has significantly broadened the scope of treatable diseases, a feat largely facilitated by the encapsulation of APIs in lipid-based nanocarriers. Presently, researchers are directing their efforts toward optimizing nanocarriers to improve their capacity for effective encapsulation, safe delivery, and targeted release. This effort demands extensive empirical work, which can be greatly supported by computational approaches. Accordingly, this review concentrates on computational techniques that furnish valuable insights into the composition screening and rational design of lipid-based nanocarriers.

### 1.1 Structure and Classification of Lipid-Based Nanocarriers

In this review, lipid-based nanocarriers (LNCs) will be categorized based on their structure into two distinct types: those with aqueous cores and those with soft cores. Both types of LNCs feature a surface layer of lipids; however, aqueous core LNCs consist of one or more lipid bilayers surrounding a water droplet, whereas soft core LNCs can possess a nonaqueous, dense, and/or multicomponent phase interior with a structure that remains fully unresolved. Liposomes are the prototypical example of aqueous core LNCs, while LNPs represent soft-core LNCs. Solid LNCs are out of the scope of this Review and were recently reviewed elsewhere.^22^

**Figure 1:**
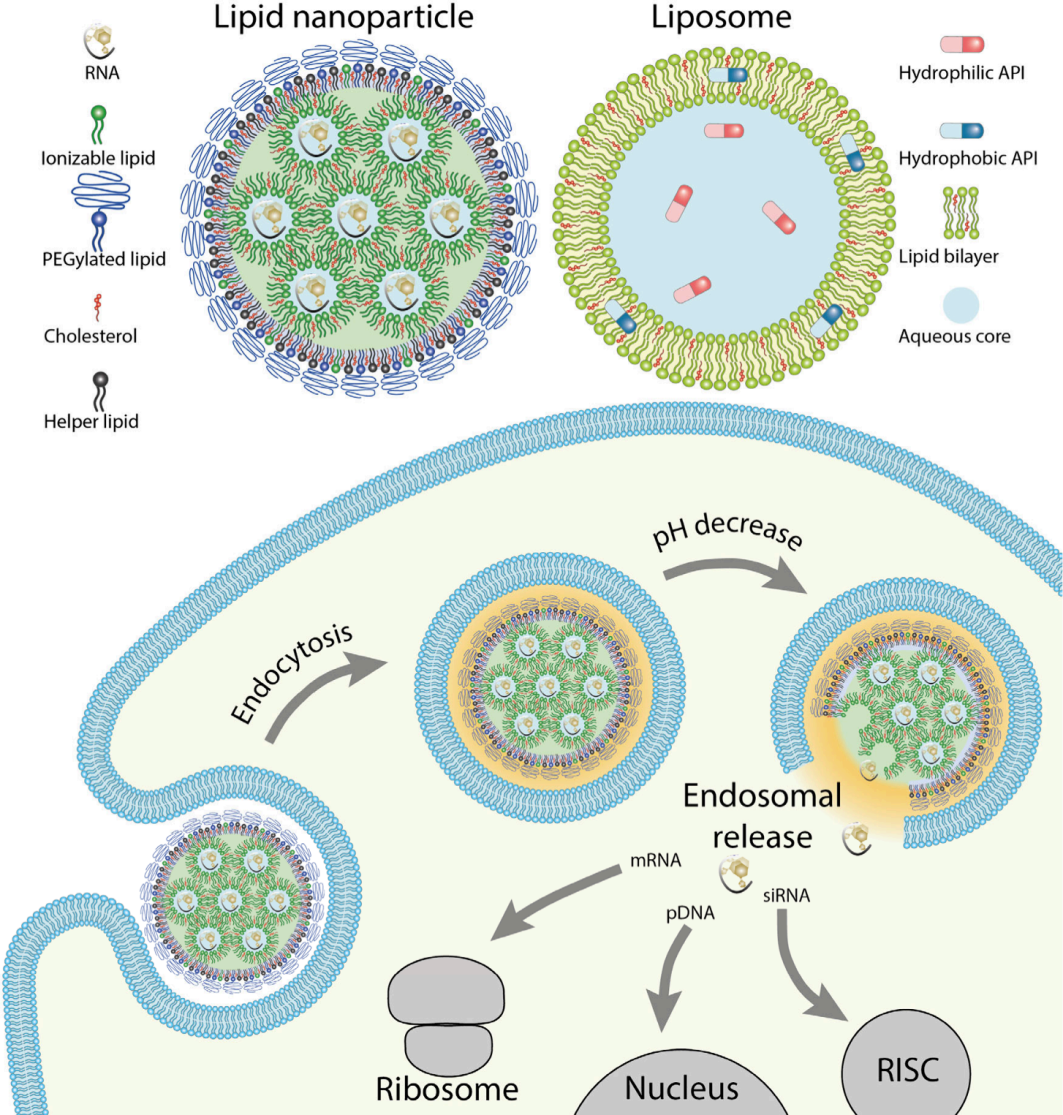
Structure of lipid nanoparticle and liposome (upper panel) and schematic of LNP endocytosis and pH-induced release. RISC stands for RNA-induced silencing complex.

Liposomes are vesicles surrounded by a lipid bilayer (Figure 1) with diameters ranging from tens of nanometers up to micrometers.^23^ Their structure offers versatility in delivering both hydrophobic and hydrophilic APIs,^7,24^ including a diverse range of therapeutic agents such as drugs, nucleic acids, and imaging agents.^25^ Hydrophobic APIs dissolve within the lipid bilayer, whereas hydrophilic APIs are loaded into the aqueous core. In the case of hydrophobic APIs, liposomes possess the advantage of facilitating API delivery through the aqueous environment.^7^ The benefits of liposomal delivery extend to both hydrophobic and hydrophilic APIs including protection from degradation, enhanced biocompatibility, improved targeting, etc.^26^

In contrast, LNPs can deliver various payloads, ranging from small drugs^27^ to peptides, proteins,^28^ imaging agents,^29^ and, most significantly, nucleic acids. The precise structure of LNP remains incompletely resolved, with current structural hypotheses proposing a phospholipid-rich monolayer enveloping the LNP core.^30−32^ The commonly accepted structural model suggests that the LNP core comprises positively charged (ionizable) lipids surrounding the nucleic acid in a lipid inverted hexagonal-like phase (Figure 1).^33,34^ However, nucleic-acid-rich cores or separate water-rich and nucleic-acid-rich regions have also been observed.^35^ Cryo-EM images have depicted onion-like multilamellar LNP structures in certain LNP formulations containing small interfering RNA (siRNA),^36^ while those containing mRNA exhibit a “bleb” phase.^37^ Though initially “bleb” was considered as lipid-bilayer-like structure surrounding water droplets,^38^ currently the presence of mRNA in the “bleb” phase is expected,^37^ though its overall effect on the RNA delivery efficiency is still discussed.^39^ The organization of the LNP core appears to be influenced by both the lipid composition and the type of encapsulated cargo.^40−43^ Both aqueous and soft-core LNCs can be composed of phospholipids or sterols commonly found in the human body. However, the enhanced functionality of current LNCs has been achieved by using functionalized artificial lipids, lipid-like molecules, or amphiphilic dendrimers.^44^

During the early stages of liposomal delivery, liposomes predominantly consisted of ‘ordinary’ lipids such as phospholipids and cholesterol. Over time, cationic lipids were introduced to enhance the loading capacity of negatively charged DNA and improve *in vitro* gene expression.^45^ However, contemporary LNP compositions are more complex. The significance of lipid composition, surface functionalization, lipid titratability, and other lipid components has been extensively reviewed elsewhere.^6,26,46^ Therefore, only the key aspects are highlighted here, including the lipid phase behavior, surface functionalization, and lipid-based responses to specific triggers.

The composition of lipid mixtures significantly influences the internal structure, rigidity, and lipid phase adopted by LNCs.^47,48^ The synthesis of a plethora of artificial lipids expanded the portfolio of possible lipid chemical nature, allowing for precise tuning of lipid shape and physicochemical properties by adjusting the amount and positioning of individual chemical groups.^49^ While lipids with a cylindrical shape tend to adopt a lamellar organization, leading to the formation of liposomes or mutilamellar LNCs, lipids with a conical shape prefer an inverted hexagonal phase, rendering them more likely to form LNPs.^50^ The conical shape is often found in lipids with small head groups and multiple lipid tails,^51^ as well as (poly)unsaturated lipids^52^ and lipidoid dendrimers.^53^ Increasing length and saturation of phospholipid chains or increasing cholesterol content enhances the ordering of the lamellar phase of LNCs, making them less permeable and reducing the loss of hydrophilic cargos during transport.^46^ Moreover, lipid mixtures with phase transition temperatures close to body temperature can facilitate temperature-triggered release^26^ induced by local overheating. The composition of lipid mixture also affects the size of resulting particles.^46^ With a wide array of natural lipids, artificial lipids, and dendrimers available, there are numerous possibilities for tailoring LNCs for specific purposes.

Regardless of their chemical composition, nanoparticles of tens of nanometers in size face rapid clearance from the bloodstream. Especially LNCs composed of lipids with permanent positive charges encounter issues such as toxicity and swift clearance.^54^ Coating their surfaces with polyethylene glycol (PEG) chains has led to particles with prolonged circulation, prevention of opsonization and later phagocytosis, and reduced aggregation.^55,56^ However, PEGylation may also cause unwanted immune reactions^57,58^ or diminish particle uptake by target cells.^59^ In organized LNCs, the PEG surface is employed by incorporation of PEGylated lipids into the surface layer of the LNC. PEG effects in LNCs are also associated with the formation of the biomolecular corona, primarily consisting of proteins that absorb onto the LNC surface.^60^ PEGylated lipids can modulate the size of LNC and its interactions with other biomolecules by the level of surface coverage.^61,62^ The chemical structure of the lipid tails of PEGylated lipid can be tailored to adjust the stability of PEG and, if desired, designed to detach from the particle over time,^63^ thereby facilitating interaction with body enzymes, antibodies, and other entities.

Lipid-based API delivery offers the ability to control API release through various chemical-physical properties, which can be considered environmental triggers.^6,55,64^ Such triggers include temperature (controlling the lipid phase),^46,65^ light (in case of presence of photoswitchable lipidoids),^66^ and pH (with lipid containing ionizable groups).^49,67^ Recent attention has been particularly focused on ionizable lipids (ILs), whose physical and chemical properties are pH-sensitive. This feature can be exploited to initiate API endosomal release (Figure 1), triggered by the natural decrease in endosomal pH during endosome maturation after endocytosis of the API-containing nanocarrier.^49,68^ In a neutral extracellular environment, ILs remain net-neutral, but they become positively charged in late endosomes. This protonation in the late endosome leads to release of the API, while reducing the unwanted toxicity associated with permanently positively charged cationic lipids.^69^

The current state-of-the-art experimental testing of APIs delivered by LNCs provides precise data regarding the delivery efficiency and concentration of APIs in various parts of the body, indicating the efficacy of these systems.^69,70^ However, uncertainty persists, regarding structural aspects. While dynamic light scattering experiments provide clear information concerning size distribution of LNPs,^71^ structural data, primarily derived from cryo-EM images, suffer from significant blurring, leading to diverse interpretations for different regions.^34,37,41,72−76^ Furthermore, a comprehensive understanding of the inherent dynamics of the system and its underlying mechanism of action is also lacking.

Enhanced load limits or delivery efficiency of LNCs can be achieved by rationally designing individual lipid molecules and their mixtures along with precise functionalization tailored for a specific API and site of action. To reach these design goals, an atomic-level understanding of the API encapsulation process, LNC structure and stability, and mechanism of API delivery is essential. However, this level of knowledge is currently beyond the reach of experimental techniques. In this context, computational tools can offer a viable alternative. Molecular dynamics (MD) simulations provide fine temporal and spatial resolution of dynamical phenomena and can offer insights into structure and mechanism of action that are unachievable by any experimental technique.^77^ The vast amount of data gathered by experiments, linking composition to structure and function, can be processed by using machine learning (ML) approaches, leading to possible virtual screening pipelines of LNC compositions. Moreover, combining data from both experiments, ML models, and MD simulations can help identify desired structure–function relationships, ultimately leading to the tailored design of LNC for individual API.

This Review focuses on computational approaches for lipid-mediated API delivery. The basic background of the computational methods is not covered, as these are discussed elsewhere.^78−81^ Instead, the use of computational tools is critically examined from two perspectives: physics-based methods, represented by MD simulations and data-based methods, ranging from QSAR to current ML approaches. The major goal of this review is to provide guidelines for setting up MD simulations and discuss the limitations of each individual approach in detail in order to improve the accuracy of the reader’s simulations and incorporate current best practices in computational methods. Additionally, the currently available data-based techniques used in lipid-mediated API delivery are explored. Finally, recent theoretical investigations of liposomal and LNP-based delivery are reviewed, and the insight they can provide into experimental observations is explained. Simple studies of permeability or partitioning are not covered, as they are reviewed elsewhere^82,83^ and fall outside the scope of this Review.

## 2 Computational Methods

Computational methods for investigation of lipid-mediated delivery systems can use two distinct approaches: physics-based methods and data-driven methods. Physics-based methods describe a real system using physical-chemical models adopting rational approximations of the structural features of the real system. They allow for the prediction of the phenomenological features of the studied system. On the other hand, data-driven methods do not need to describe the underlaying physical-chemical features of the investigated system, but they identify the structure–property relationships based on available empirical data collected for investigated and related systems. These two approaches are not mutually exclusive but can complement each other, enhancing the overall utility and effectiveness of the outcome.

### 2.1 Physics-Based Methods

**Figure 2:**
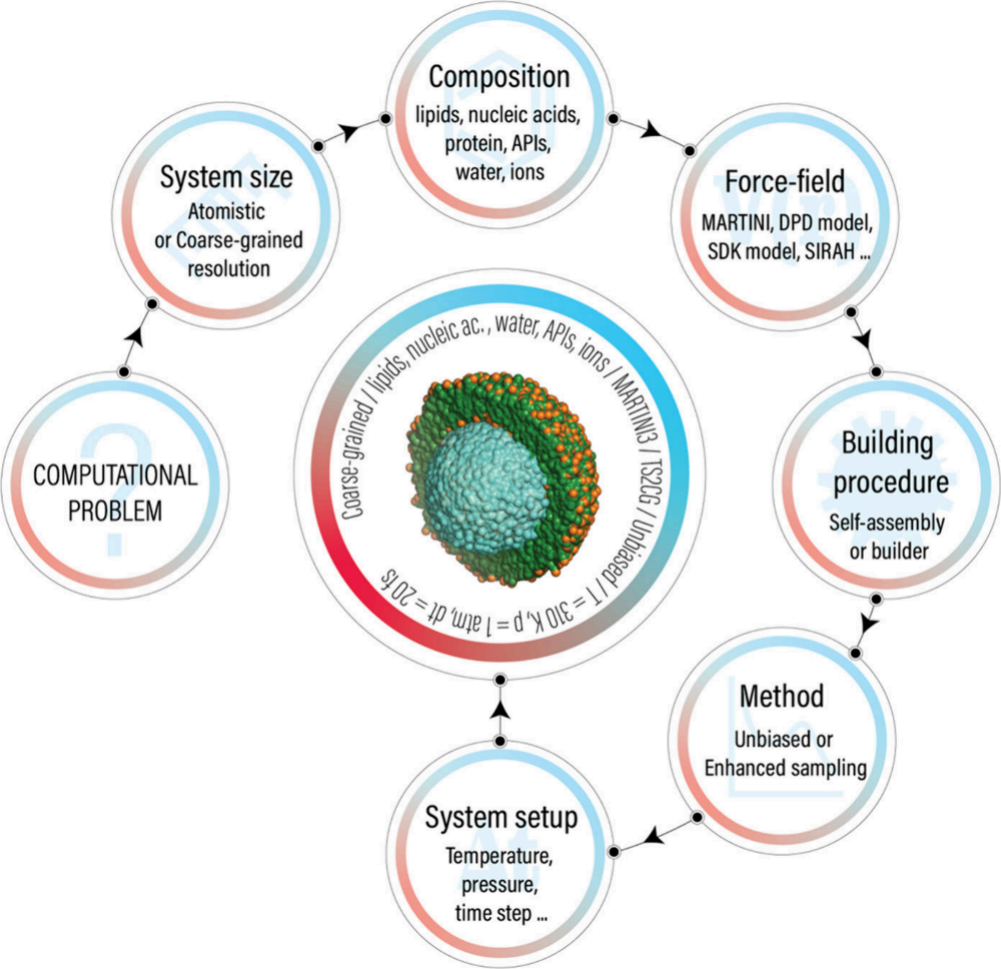
Schematic representation of individual choices underlying the computational design of liposomal systems. This workflow represents individual steps of liposomal system preparation starting from the choice of the resolution (AA or CG) through selection of individual chemical components comprising the system up to the choice of FF, building procedure (using builder or self-assembly) and finally to the setup of simulation conditions.

Classical MD simulations serve as a common physics-based method for exploring LNCs. Classical MD simulations utilize molecular mechanics for evaluation of the potential energy and its derivatives. Potential energy is enumerated using empirical force fields (FF), which contain a set of equations and empirical parameters describing both bonded and nonbonded interactions. Execution of an MD simulation seems to be a straightforward task (particularly when assisted by user-friendly software), but its setup requires careful consideration of several critical aspects, such as the choice of resolution, FF, environmental conditions, structural features, and simulation approach (Figure 2, Box 1). The review does not contain fundamentals of molecular modeling which can be found in the literature.^78−81^ However, in the subsequent parts of this section, guidance for making informed decisions concerning the MD setup is offered.

Box 1 **Critical Steps for Simulation Setup**

***Resolution.****The choice of resolution is always a compromise among system size, targeted time scale, and available computer resources.* All-atom (AA) FFs describe every single atom, offering atomic resolution of individual interactions. However, their computational demands limit their application to models containing fewer than ∼10^7^ atoms, constraining their effectiveness to scales approaching ∼30 nm. While it is feasible to construct such larger models, contemporary computer power limits the feasibility of conducting multimicroseconds simulations on such scales and the dynamical processes can usually be captured in scales until ∼10 nm. Coarse-grained (CG) FFs simplify the studied molecules, significantly reducing the computational costs allowing for the aspiration of simulating entire cells.^78,84^ However, this comes at the expense of losing fine atomistic resolution.

***Force Field.*** LNCs particularly with API and in a biological environment represent a complex molecular system comprising various components. *Each component must be well and completely described by the FF and the used FFs must be mutually compatible.* In the case of missing parameters, they must be developed and carefully tested. The choice of FF is a delicate task as it can critically impact the observed results and even lead to biased results.

***Environmental Conditions***.**** The chemical and biologically relevant conditions of a phenomenon of interest must be considered carefully. Although some simplifications are often required in MD simulations, it is desirable to reproduce the experimental conditions as accurately as possible. Consequently, when setting up a simulation, *all relevant factors such as temperature, ionic strength, pH, etc., should be considered*. For instance, the targeted pH defines protonation states of titratable groups. Inaccuracies in representing protonation states or lipid phase can potentially result in modeling entirely different phenomenon than originally intended.

***Initial Configurations and Structural Features*****.***Classical unbiased MD simulations sample the configuration space locally*. This means that systems may not deviate in their structures far from the starting configuration during the classical MD simulation. This calls for particular attention to the choice of starting structure, especially in the case of all-atom MD simulations, where sampling can be computationally expensive.

***Time Scale.*** As previously mentioned, classical unbiased MD simulations sample locally and can be linked to a single molecule experiment. This implies that *the simulation time scale must be considerably longer than the time scales of the studied phenomena*. Otherwise, the probability of observing the studied phenomenon in a single MD simulation is very low. The probability can be enhanced by performing multiple replicates of the same system. If targeted biologically relevant time scales are longer than the simulation time scale, then dedicated enhanced sampling methods can also be employed to address this challenge.

#### 2.1.1 All-Atom Resolution

AA FFs are well established tools for simulating lipid-mediated delivery systems.^85,86^ In standard AA FFs, all atoms in the system are represented by van der Waals particles with partial charges, connected by bonds based on the system topology (structural formula). FF parameters are optimized to reproduce experimental observables and structural properties of the studied systems.^80^ The choice of AA FFs must be done carefully to ensure accurate reproduction of experimental observables, crucial for capturing the interaction between cargo molecules, and provide insight into stability of API-lipid complexes, the efficiency of delivery systems, and the mechanisms underlying API release or uptake.^86^ The next section reviews the state of the art of the most popular AA FFs used in biomolecular simulations relevant to lipid-based delivery systems, which include widely used FFs from CHARMM, AMBER, and OPLS FF families. Additionally, united atoms GROMOS force field, which has been successfully employed in studies of lipid systems,^87−89^ is also worth mentioning, although its applications to LNCs have been limited. The focus is only on the current most advanced version of FF and the development of individual FFs is provided in Table 1.

**Table 1:** Development of Classical All-Atom Force Fields^a^

	Name	Lipid types	ref	Notes
AMBER	LIPID11	PC, PE, PG, PS, PI, PA, CHOL	Skjevik et al.,^90^ 2012	Surface tension needed, heads and tails as separate residues - plug and play approach
	GAFFlipid	PC, PE	Dickson et al.,^91^ 2012	LJ params revisited, lipid molecule as one residue, tensionless simulation
	Lipid14	PC, PE	Dickson et al.,^92^ 2014	LJ, torsion, partial charged revisited, plug and play
	Lipid17	PC, PE, PS, PG, PA, CHOL		Released with Amber17^93^
	Lipid21	PS, PG, PA, PUFA, SM	Dickson et al.,^94^ 2022	Head group and acyl chains torsion parameters
	Slipids	Saturated PC	Jämbeck et al.,^95^ 2012	Based on ab initio calculation, bulk alkane liquids properties
	Slipids	Unsaturated PC, PE	Jämbeck et al.,^96^ 2012	Tested compatibility with protein FFs ff99SB, ff99SB-ILDN, and ff03
	Slipids	PG, PS, SM, CHOL	Jämbeck et al.,^97^ 2012	Water permeability validated
	Slipids		Grote et al.,^98^ 2020	Head group dihedral parameters
	Slipids	DLin-MC3-DMA	Ermilova et al.,^99^ 2020	MC3 PMF across helper lipid bilayers
	Slipids	SM-102, ALC-0315	Ermilova et al.,^100^ 2023	PMFs across various PC bilayers
CHARMM	CHARMM22	PC, PE	Schlenkrich et al.,^101^ 1996	Fixed surface area needed
	CHARMM22	unsaturated tails	Feller et al.,^102^ 1997	Fixed surface area needed
	CHARMM27	PC, PE, SDS	Feller et al.,^103^ 2000	Aliphatic tails, fixed surface area/surface tension needed
	CHARMM27r		Klauda et al.,^104^ 2005	Torsions of aliphatic chains
	CHARMM36	PC, PE	Klauda et al.,^105^ 2010	Headgroup torsional parameters, LJ and partial atomic charges of the ester linkage, tensionless simulation
	CHARMM36c	CHOL	Lim et al.,^106^ 2012	
	CHARMM36	PIPs	Wu et al.,^107^ 2014	Membrane builder added to CHARMM-GUI
	CHARMM36	PS, SM	Lee et al.,^108^ 2016	Input generator for multiple simulation packages added to CHARMM-GUI
	CHARMM36	Glycolipids, lipoglycans, cardiolipins	Lee et al.,^109^ 2019	Glycolipid builder added to CHARMM-GUI
	CHARMM36	ILs, PEGylated lipids	Park et al.,^110^ 2021	ILs and PEGylated lipids added to CHARMM-GUI
	CHARMM36	Functionalized sterols, other phospholipids	Pogozheva,^111^ 2022	18 complex biomembrane systems added to CHARMM-GUI
OPLS	OPLS-AA	DPPC	Maciejewski et al.,^112^ 2014	Reparameterization of the OPLS-AA FF to improve lipid properties
	OPLS-AA	Tails for PC, CHOL	Kulig et al.,^113^ 2016	Unsaturated tails, validated CHOL parameters, data provided in^114^
	OPLS-AA	PEG	Stepniewski et al.,^115^ 2011	Effect of PEGylation on lipid bilayer is investigated
	OPLS-AA	DSPE-PEG	Magarkar et al.,^116^ 2014	DOXIL composition
	OPLS-AA	PE, vinyl	Rog et al.,^117^ 2015	Effect of plasmalogens is investigated
	OPLS-AA	PS, SM	Rog et al.,^118^ 2016	Interdigitation studied
	OPLS3e	POPC	Kurki et al.,^119^ 2022	Verifying OPLS3^120^ accuracy for a POPC system, part of Schrodinger software
	OPLS-AA	CHLS, CHLSa	Mahmoudzadeh et al.,^121^ 2021	Cholesteryl hemisuccinate in neutral and ionized form
	OPLS/2020		Jorgensen,^122^ 2023/2024	Unsaturated hydrocarbons, alcohols and ethers
	OPLS4		Lu et al.,^123^ 2021	Novel strategy for charged species

a Lipid force fields or their versions (Name) are sorted according to the FF family, major lipid types introduced in the respective publication, and notes relevant for the publication.

##### 2.1.1.1 AMBER FF Family

The AMBER (Assisted Model Building and Energy Refinement) FF family, initially developed by the Kollman group, is a comprehensive suite of empirical potentials designed for biomacromolecular simulations.^124^ Originally created as one of the first atomistic FFs for simulating nucleic acids and proteins,^125^ AMBER has since evolved into a highly versatile FF families with a broad range of applications for simulations in the condensed phase.

A key innovation of AMBER’s parametrization procedure is the use of fixed partial charges assigned to atomic centers, determined by the Restrained Electrostatic Potential (RESP) method.^126^ Over time, this methodology has been extended to encompass a wide variety of molecular species, including proteins, nucleic acids, carbohydrates, and lipids, each tailored with specific parameters for enhanced modeling accuracy. AMBER’s development remains a collaborative effort, ensuring these tools stay at the cutting edge of MD simulations. The current ff19SB version^127^ is particularly effective for modeling peptides and proteins. Likewise, GLYCAM FFs series provides parameters for a large set of carbohydrates.^128^

Lipid FF for the AMBER family are based on a flexible, modular framework and parametrization strategy.^90^ This strategy employs the AMBER RESP procedure for deriving fixed partial atomic charges, combined with a plug-and-play approach for incorporating lipid head groups and tails, enabling the creation of various phospholipids. The latest version, Lipid21,^94^ allows tensionless simulations, accurately reproduces NMR headgroup parameters and phase transition temperature. Apart from PC, PE, and cholesterol, Lipid21 also supports anionic lipids (phosphatidylglycerols (PG), phosphatidylserines (PS), and phosphatidic acids), polyunsaturated fatty acids (PUFA), and sphingomyelin (SM). AMBER’s modular strategy ensures compatibility with other AMBER-based components like small molecules, peptides and proteins, carbohydrates, and nucleic acids.

The Slipids (Stockholm Lipids)^95^ FF is derived from CHARMM FF nomenclature but follows the AMBER FF philosophy, ensuring compatibility with AMBER protein FF family like ff99SB, ff99SB-ILDN, and ff03.^96^ Slipids was based on the correct representation of thermodynamic properties of the hydrocarbon tail analogues, such as alkane heats of vaporization and densities. Currently, it allows tensionless simulations of various phospholipids, cholesterol and sphingomyelin,^97^ correctly representing the membrane structural parameters.^98^ Ionizable lipids, such as DLin-MC3-DMA,^99^ SM-102, and ALC-0315,^100^ were added recently, though these showed inconsistencies with neutron reflectometry data, failing to reflect bilayer thickness or phase separation at neutral pH.^32^

In the AMBER family of FFs, DNA and RNA parameters are developed separately. There are three main branches of AMBER DNA FFs: BSC0 and BSC1,^129^ developed by Orozco group, OL15^130^ and OL21 from a collaborative effort,^131^ and Tumuc1^132^ by Liebl and Zacharias. OL21 and Tumuc1 have advanced in simulating double-stranded DNA,^133^ with OL21 showing improved accuracy for Z-DNA.^133^ Modern RNA FFs include χOL3,^134,135^ AMBERTOR,^136^ AMBER_DES_,^137^ and one introduced by Chen and Garcia.^138^ χOL3 reparametrizes the glycosidic dihedral potential for nucleotides to prevent untwisted ladder-like A-RNA structures,^134^ making it highly validated, through it needs improvement for description of short single-strand tetranucleotides,^139^ UNCG tetraloops,^139^ and observed over compaction of single-stranded RNAs.^140^ The χOL3 shortcomings can be mitigated using a straightforward potential function known as gHBfix.^141−143^ AMBERTOR reparametrizes all dihedrals but distorts A-RNA duplexes,^144^ while AMBER_DES_ includes reparameterizations in both dihedral and nonbonded terms but tends to overfit to the A-form.^145^ Chen and Garcia’s FF modifies nucleobase van der Waals parameters, allowing greater flexibility,^146,147^ but it still shows overstabilization of base pairing^146^ and an imperfect description of A-RNA double helix structures.^148^

To incorporate additional molecules such as small APIs, into AMBER FFs, atomic types, bonding and nonbonding parameters, and partial charges must be assigned via the RESP procedure.^126^ General AMBER FF (GAFF2)^149,150^ is designed for describing a variety of molecules in terms of their bonded and nonbonded parameters. This is usually performed by freely available Antechamber from AmberTools. For uncommon molecules, vibrational analysis may be needed to obtain bond parameters,^151^ and in some cases, potential energy scan is required for dihedral angles, particularly with conjugated bonds. This method is compatible with AMBER family lipid FFs.^152^ Lipid-like molecules can use Lipid21, which offers optimized atom types and bonded parameters. For hybrid lipids, different FFs may be combined; for example, nonlipid parts like PEG can use GAFF2, while carbohydrates can be handled by GLYCAM FF.^128^ The linker between these two parts requires careful parametrization. Generally, it is advised to verify FF compatibility in systems modeled with different AMBER FF subfamilies by comparing parametrization procedures and validating with experimental data whenever possible.

##### 2.1.1.2 CHARMM FF Family

The CHARMM (Chemistry at HARvard Macromolecular Mechanics)^153^ AA FF is widely used for biomolecular simulations, including lipids, proteins, nucleic acids, and carbohydrates. It has been extended to cover drug-like molecules through the CHARMM General Force Field (CGenFF),^154^ which is compatible with additive biomolecular FFs. CHARMM parameters are optimized using small molecules and macromolecular data from quantum mechanical calculations and experimental measurements. Point charges are determined to accurately replicate quantum mechanical interaction energies and geometries of model compounds, involving water molecules.^155^

The current lipid FF CHARMM36^105^ represents well the bilayer surface tension and deuterium order parameters, including various phospholipids and cholesterol. CHARMM-GUI builder expanded CHARMM36 to include cationic ionizable and PEGylated lipids, accurately reproducing experimental reflectivity profiles.^32,110^ Various lipid types were integrated into the CHARMM-GUI Input generator,^108^ including glycolipids, lipoglycans and peptidoglycans,^109^ cardiolipins and phosphoinositide,^107^ and other biomembrane lipids.^111^ Although the parametrization details for many new lipids remain unspecified in recent publications, fragment-based approach likely underpins these additions, creating the richest biomolecular library among the available FFs. This expanded range of input parameters and compatible molecular models, especially in CHARMM36^156^ simplifies complex biosystem simulations, including realistic biomembranes,^111^ without the need for intricate parametrization of individual components.

The CHARMM FF is widely used for nucleic acid simulations. CHARMM22^157^ and CHARMM27^158^ have been refined to the latest version, CHARMM36,^159^ which focuses on improving DNA simulations by adjusting ε/ζ dihedrals, particularly for BI/BII B-DNA states.^159^ However, CHARMM has shown lower structural stability in B-DNA compared to recent AMBER FFs^160,161^ and reduced stability in G4 simulations.^162,163^ CHARMM36 was also optimized for RNA by adjusting the ribose hydroxyl dihedral potential,^159^ but some instabilities persist, leading to fraying within base-paired segments.^164^

Incorporating xenobiotics and other molecules not covered by CHARMM36 is straightforward due to its fragment-based approach and the ability to parametrize additional molecules via CGenFF.^154^ It is generally not recommended to rely on quantum mechanical calculations for this purpose. Instead, users are advised to use CGenFF tool,^165^ either independently or through the CHARMM-GUI.

##### 2.1.1.3 OPLS FF Family

The OPLS (Optimized Potentials for Liquid Simulations)^166,167^ force field, originally designed for small organic molecules, has been extended for the accurate modeling of proteins, nucleic acids, and lipids. OPLS parameters are derived from fitting to experimental and quantum mechanical data, including heats of vaporization, densities, and vibrational frequencies.^167^ Different versions, such as OPLS-AA, OPLS-UA, and OPLS3e, provide varying levels of detail and accuracy for simulating diverse biomolecular systems.

Lipid parameters for OPLS-AA were developed later than for CHARMM or AMBER, including, PC, PE, SM, and cholesterol.^113,117,118^ OPLS FFs have been widely used for studies of PEGylated lipids.^116,121,168−172^ Current development of OPLS does not focus on lipids directly but can derive parameters from the general parametrization approach. OPLS development has split into two branches, academic OPLS/2020^122^ and OPLS4,^123^ part of Schrödinger suite.

For nucleic acids, OPLS-AA was reparametrized to create OPLS-AA/M, with adjusted β, χ and γ dihedral angles, methyl phosphate, phosphodiesters, ribose, and carbohydrate torsion parameters.^173^ Further refinements improve RNA simulations by reparametrizing α and γ dihedral angles,^174^ enhancing the accuracy for short sequences or noncanonical RNA motifs, though challenges persist for tetraloop-type structures and larger systems. OPLS-AA/M remains fully compatible with the original version for proteins and small molecules.^174^ The OPLS FF can also be applied to study nucleic acid interactions with lipids, such as in lipid bilayers or micelles.^175^ OPLS-AA lacks updated DNA parameters, making it less ideal for DNA-based simulations.

To add new small xenobiotic molecules into OPLS, the recommended approach is to utilize the LigParGen Server,^176^ which generates OPLS-AA parameters directly.

##### 2.1.1.4 Polarizable Force Fields

Polarizable FFs have emerged as powerful tools in molecular simulations, offering enhanced accuracy in modeling complex molecular interactions compared to traditional nonpolarizable FFs. By explicitly considering electronic polarization, polarizable FFs provide a more realistic representation of molecular systems,^177,178^ especially those involving charged species like nucleic acids and lipids.^179−181^ Options like AMOEBA (Atomic Multipole Optimized Energetics for Biomolecular Applications)^182^ and CHARMM Drude Polarizable Force Field^183−185^ are available. Unlike nonpolarizable FFs, which use fixed atomic charges, polarizable FFs include parameters for polarization-induced dipole and may better capture properties like p*K*_a_.^186^

A widespread use of polarizable FFs faces challenges in parametrization^187^ and performance, requiring more computational resources due to additional parameters and enumeration of polarization effects. MD simulations with polarizable FFs heavily depend on the used code. The AMOEBA model was recently implemented into the Tinker-HP package with GPU acceleration,^188^ while Drude2023 in OpenMM,^189^ with GPU acceleration, can outperform classical MD with PME.^190^ However, our ongoing work shows a significant performance drop using Drude FF in OpenMM compared to classical FFs in AMBER. Despite these challenges, polarizable FFs hold promise in advancing biomolecular systems, offering insights into biological molecules’ atomic-level behavior. For more details on polarizable FFs, explore the comprehensive review provided by Jing et al.^191^

#### 2.1.2 Coarse-Grained Resolution

To address sampling limitations in atomistic MD simulations, researchers often use coarse-grained (CG) models. CG models reduce the number of particles, enabling the study of larger systems on more realistic scales. With a smoother energy landscape and longer integration time steps, they allow for investigating phenomena over longer time scales (up to tens of milliseconds), insights into molecular features and energetics essential for understanding mesoscopic and macroscopic behavior. While AA MD simulations have evolved since the 1970s, CG models, like Warshel and Levitt’s protein model,^192^ gained significant momentum only in the 2000s.

In CG models, multiple atoms are grouped into beads, reducing degrees of freedom and enabling simulations at larger spatial scales (typically up to 100 nm). The number of atoms per bead varies, typically ranging from 2 to 10 heavy atoms. These models are parametrized to retain key system features, depending on the scientific questions and available reference data and can be derived by using either top-down or bottom-up approaches. Top-down methods aim to reproduce experimental macroscopic properties, while bottom-up approaches focus on reproducing microscopic statistics from reference atomistic simulations.^193^

One drawback of CG resolution is the loss of fine resolution, potentially leading to inaccurate representations of hydrogen bonds and other subtle structural features. CG approaches also face challenges with interpreting real time scale, entropy-enthalpy compensation issues, transferability, and temperature dependence.

Furthermore, while earlier CG models sometimes faced difficulties in maintaining certain bilayer properties under certain conditions,^194−197^ being rather qualitative than quantitative in their predictions, recent advancements have significantly improved their accuracy.^198^ Hence, for complex LNP structures, the primary challenge often lies in building a stable initial configuration. This can be addressed using the tools described in section 2.1.4. Supra-CG Models provide even greater simplification, describing larger molecular assemblies or cellular structures with fewer CG beads. While this allows simulations of complex biological processes like cellular signaling or membrane fusion on realistic time scales, it further reduces the chemical detail, limiting their applicability in studies requiring precise molecular interactions.

In summary, CG modeling offers a valuable approach for studying lipid-mediated delivery systems and other complex biological phenomena, balancing system size, length scale, and chemical detail. While CG models enable simulations at spatial and temporal scales larger than those of atomistic models, users must weigh trade-offs in resolution and accuracy. For LNCs, pragmatic CG models,^199,200^ those retaining some chemical specificity and partially based on top-down experimental approaches, are typically used. Bottom-up strategies like force-matching^201,202^ and iterative Boltzmann inversion^203^ methods and a graph-based approach^204^ are widely applied to nucleic acids^205,206^ and lipid systems,^193,207−209^ but LNCs applications are still emerging.^210^ The following section focuses on the most used pragmatic CG models for studying lipid-based systems (Table 2).

**Table 2:** Development of Coarse-Grained Force Fields^a^

Name		Lipid types	Reference	Notes
MARTINI	Martini 1	PC, PE	Marrink et al.,^211^ 2004	1st Martini model
	Martini 2	PC, PE and CHOL	Marrink et al.,^212^ 2007	Additional bead types and S-size, allowing diversity of lipid models
	Martini 2	Cardiolipins	Dahlberg et al.,^213^ 2007	1st cardiolipin model
	Martini 2	PEGylated lipids	Lee et al.,^214^ 2011	1st PEGylated lipid model
	Martini 2	Glycolipids and PIPs	López et al.,^215^ 2013	1st Glycolipids and PIPs
	Martini 2	PC, PE, PG, PS and PA	Wassenaar et al.,^216^ 2015	The insane tool for generating custom membranes was released
	Martini 2	Sterols and hapanoids	Melo et al.,^217^ 2015	Improved CHOL in additional to new sterols
	Martini 2	Lipopolysaccharides	Van Oosten et al.,^218^ 2016; Hsu et al.,^219^ 2016; Ma et al.,^220^ 2017	Extension to bacterial membrane lipids
	Martini 2	Glycolipids	Gu et al.,^221^ 2017	Improved glycolipid parameters for Martini 2
	Martini 2	Phospholipids	Carpenter et al.,^222^ 2018	Improved phospholipid parameters for Martini 2
	Martini 2	PEGylated lipids	Grünewald et al.,^223^ 2018	Improved and transferable PEG model, including PEGylated lipids
	Martini 3	Phospholipids	Souza et al. et al.,^224^ 2021	Release of the new Martini 3 interaction matrix
	Martini 3	PIPs	Borges-Araújo et al.,^225^ 2021	Improved parameters of PIP headgroups for Martini 3
	Martini 3	Glycolipids	Grünewald et al.,^226^ 2022	Parameters for Glycolipid headgroups
	Martini 3	CHOL	Borges-Araújo et al.,^227^ 2023	Release of the Martini 3 Cholesterol model
	Martini 3	Lipopolysaccharides	Vaiwala et al.,^228^ 2023; Brandner et al.,^229^ 2024	E-Coli Lipopolysaccharide
	Martini 3	ILs, sterols and PEGylated lipids	Kjølbye et al.,^230^ 2024	Parameters for several lipids found in LNPs
	Martini 3	Full lipidome library	Pedersen et al.,^1000^ 2025	Expansion of Martini 3 lipid library, with tails differing by two carbon atoms
SDK/SPICA	SDK	PC and PE	Shinoda et al.,^231^ 2010	1st SDK model
	SDK	CHOL	MacDermaid et al.,^232^ 2015	Introduction CHOL in SDK
	SDK	Free fatty acids and ceramides	MacDermaid et al.,^233^ 2020	Observation of inverse-hexagonal phases
	SPICA	CHOL, PC, PE, PS, PG, Sphingomyelin	Seo et al.,^234^ 2019	Domain formation induced by cholesterol is validated
	pSPICA	PC, PG and PE	Miyazaki et al.,^235^ 2019	Incorporation of a polar water model
SIRAH	SIRAH	DMPC	Barrera et al.,^236^ 2017	Implementation of lipid models on SIRAH
	Fat SIRAH	PC, PE, PS	Barrera et al.,^237^ 2019	PC, PE and PS head groups; MY, PA and OL tails
DPD	DPD	PE	Groot et al.,^238^ 2001	First parameters for a phospholipid
	DPD	DMPC	Kranenburg et al.,^239^ 2004	Improved phospholipid parameters
	DPD	DMPC	Gao et al.,^240^ 2007	Improved force parameters for simulating lipid bilayers in water
	DPD	PC	Li et al.,^241^ 2016	Models for simulating with explicit solvent
	Im-DPD	DMPC, DMPG, DPPC and DOPC	Wan et al.,^242^ 2018	Models for simulating with implicit solvent

a Lipid force fields or their versions (Name) are sorted according to the FF family, major lipid types introduced in the respective publication and notes relevant for the publication.

##### 2.1.2.1 Martini Model

The Martini FF, pioneered by Marrink et al.,^211,212^ stands as a cornerstone in CG simulations, particularly for biomembranes.^243^ First introduced in 2002, this FF has undergone several critical reviews^244,245^ and improvements, with the latest version, Martini 3, released in 2021 by Souza et al.^224^ Martini employs a modular design where beads represent four, three, or two non-hydrogen atoms. The model uses a top-down strategy based on thermodynamic data for the nonbonded interactions and a bottom-up approach for bonded interactions. This allows for the straightforward parametrization of various lipid types, including specialized lipids such as glycolipids,^215,221,226^ PEGylated lipids,^214,223^ cardiolipins,^213,246,247^ and sterols,^217,227^ facilitating the simulation of complex membranes with realistic lipid compositions.^198,248,249^ More recently, an extensive library for ionizable lipids has also been released^230^

The Martini FF also covers proteins,^250,251^ carbohydrates,^226,252^ nucleotides^253,254^ and polymers,^255^ allowing for the study of diverse membrane-related processes. While solvent is typically treated explicitly, an implicit solvent version called “Dry” Martini,^256^ is also available. For processes that require long-range electrostatic interactions, polarizable water and ion models have been developed.^257−259^ The integration of the titratable water model into Martini 3,^260^ along with the implementation of lambda-dynamics,^261^ enables advanced constant pH simulations.

One of Martini’s biggest strengths is its expanding model library.^262^ The latest iteration, Martini 3,^224^ greatly increased the discrimination of chemical space by increasing the number of bead types and allowing better overall packing of molecules with finer mappings, resulting in greater accuracy and flexibility. Beside lipid systems, Martini has also been extensively employed to simulate various biological systems, encompassing membrane proteins, lipid bilayers, and nucleic acids (reviewed in Marrink et al.^243^) and is implemented in major simulation packages such as GROMACS,^263^ OpenMM,^189,264^ NAMD,^265^ Materials Studio, and LAMMPS.^266^

However, the Martini FF currently cannot model large conformational changes and folding events of protein and nucleic acids. To address these limitations, Go̅Martini models were introduced, enabling the sampling of large conformational changes and correcting environmental bias when simulating proteins.^267−269^ A further limitation of Martini 3 is the gradual incorporation of specific molecular classes. Currently, users wanting to perform simulations with nucleic acids or nanomaterials must either use Martini 2 or prepare topologies from scratch.

##### 2.1.2.2 SDK/SPICA Model

The SDK (Shinoda–DeVane–Klein) Model^231,270^ employs a 3-to-1 mapping, sharing similarities with Martini models. The SDK FF utilizes soft interaction potentials, enabling good reproduction of heats of vaporization and surface tensions. It prioritizes thermodynamic accuracy, making it particularly suitable for studying lipid monolayers and vesicles.

In SDK simulations, the solvent is typically treated explicitly, enhancing the realism of the simulated hydrodynamic and thermal properties. However, the model lacks inherent polarizable water and ion models. Unlike Martini’s building-block approach, SDK beads are typically parametrized to represent specific groups in a molecule, allowing for tailored parametrization but requiring more effort compared to a building-block approach.

While the original SDK model accurately described lipid membrane properties,^231,232^ it struggled to represent cholesterol domain formation. A recent extension of the SDK Model rebranded as SPICA^234^ (Surface Property fitting Coarse graining) FF improved this by refining the parameters for cholesterol and various lipid types, enhancing the depiction of membrane-related phenomena. The updated SPICA model now also includes parameters for ions,^271^ proteins, and peptides^272,273^ but lacks those for RNA and DNA, limiting its use in simulating nucleic acid-related systems.

Applications of the SDK/SPICA Model span across various areas, including studies on lipid monolayer phase behavior,^231^ vesicle formation,^231^ vesicle–vesicle interaction,^274^ membrane partitioning of fullerenes,^275^ and research on lipid droplet formation.^276^ However, the SDK Model has a limited number of available lipid parameters and is currently only implemented in the LAMMPS software package.^266^

##### 2.1.2.3 SIRAH Force Field

The SIRAH^277^ (South-American Initiative for a Rapid and Accurate Hamiltonian) FF, developed by Pantano and colleagues, offers a top-down CG approach for modeling proteins and DNA.^278^ It features a mapping scheme that includes explicit solvent and ion representation and incorporates electrostatic interactions explicitly, allowing detailed treatment of charged residues and ions within simulations. However, the absence of polarizable water and ion models can limit its accuracy in environments where polarization effects are significant.

One distinctive feature of the SIRAH FF is its capability to sample conformational changes of proteins, which is attributed to its higher resolution of the peptide backbone. This allows the study of protein dynamics and some limited structural transitions but not protein folding. Despite having a comprehensive set of pre-existing molecular topologies, creating new molecules can be challenging due to the user-dependent nature of the parametrization process. Recently, the SIRAH Force Field has been expanded to include several lipid species,^237^ broadening its applicability to simulate structures such as the Zika Virus virion^279^ and SARS-CoV-2 membrane proteins.^280^ This broadens its applicability to studies related to cell membrane dynamics. Another advantage is its compatibility with both GROMACS,^263^ and AMBER software platforms,^281^ providing researchers flexibility in computational simulations.

##### 2.1.2.4 Dissipative Particle Dynamics Models

Dissipative particle dynamics (DPD) was introduced by Hoogerbrugge and Koelman in the early 1990s as a method for simulating complex fluids by incorporating hydrodynamic interactions.^282^ Groot and Waren refined the approach in 1997, establishing a framework for simulating soft matter systems.^242,283^

The level of granularity in DPD models is similar to that of recent Martini-type mapping, providing an effective approach for studying biomolecular systems at a reduced resolution. Originating from hydrodynamics principles, DPD models approximate a system of dissipative particles moving according to fluid dynamics while incorporating thermal fluctuations. By explicitly modeling solvent particles, DPD accurately captures hydrodynamic interactions and solvent-mediated effects. While standard DPD models do not include polarizable water and ion models for handling long-range electrostatic interactions, polarizable water models have been developed.^284^ A notable feature of DPD is the use of soft potentials, which enhance diffusion by smoothing the energy landscape for particle interactions.^285^ While these soft potentials can improve the accuracy of simulations, they may also introduce some unphysical phenomena such as molecular overlap. DPD models offer valuable insights into the collective behavior of lipid membranes and other soft matter systems.

Models can be extended to include RNA and DNA, although these models are not as widespread as those for lipids. DPD models offer a degree of flexibility and customization in parametrization, similar to other CG models. Strategies to construct DPD models of proteins from other CG models have also been implemented,^286^ with a primary focus on proteins and peptides.

DPD has been widely applied to investigate lipid behaviors, including cholesterol’s impact on bilayer structure,^287^ the adhesion, intake, and release of nanoparticles by lipid bilayers,^288−291^ and fusion mechanisms.^292^ Additionally, they have been employed to investigate PEG-containing polymers^293,294^ or dendrimers, further expanding the understanding of lipid interactions and behavior.^295^ However, the usage of DPD may be limited due to its primary implementation within the LAMMPS software package.^266^

#### 2.1.3 Environmental Conditions

Lipid-mediated API delivery systems are complex and highly sensitive to environmental conditions, particularly temperature. Along with the lipid composition, the temperature governs the phase behavior. Modeling biologically relevant systems requires precise temperature control to reflect the conditions of the phenomenon under investigation, which may vary depending on modeling context, such as encapsulation, administration in a body, etc. While models often simplify lipid composition, membrane complexity should be preserved^111^ to reflect all biologically relevant types of lipids. Failure to model the lipid composition accurately may impact the preferred lipid phase and the corresponding behavior.

Environmental pH is also critical, especially for the behavior of titratable groups.^49,296^ ILs have a p*K*_a_ near neutral pH^67^ so they should be protonated at acidic pH and deprotonated at neutral pH. The proper ratio of protonated to deprotonated ILs has yet to be fully clarified, but MD simulations can be used to study behavior at various pH values, as ILs can (and should) be added in a protonation state relevant to the studied environment.^297,298^ The API’s protonation state should also be evaluated based on environmental pH, which may differ between aqueous and membrane environments.^299,300^ Simulating molecules without considering the pH effect may result in an inaccurate system description.

The strength of the MD simulations lies in their ability to manipulate factors such as the composition, temperature, and protonation state. Monitoring system responses to environmental changes helps to study cargo release under specific triggers and uncover the underlaying mechanisms. The system can be changed either gradually (e.g., heating or adding/changing single molecules) or stepwise (e.g., changing the temperature or rapidly altering its composition). Such simulations can facilitate understanding the role of the titratable switches in lipids or individual molecular components in the efficiency of API delivery within the body.

#### 2.1.4 Building Protocols

Due to the limited time scales of simulations, considerable effort has been devoted to generating starting structures for simulations that closely mimic equilibrium states. Though random mixtures in solution could lead to equilibrated bilayers, self-assembly simulations require higher sampling and do not allow for full control over the resulting organization. For complex membranes or basic mixtures containing membrane proteins, achieving equilibrated starting structures still requires microseconds or longer, which is crucial for obtaining meaningful simulations. Therefore, for large or complex systems, ensuring proper presimulation organization is critical. This remains an area of ongoing development, with already several established codes that can facilitate this process (Box 2). Most of these codes are available as open source, allowing researchers to expand them by introducing new lipids and building blocks or modifying the code building protocols for AA (or other) resolutions. These protocols can also be combined to build complex systems with different lipid-based components such as bilayers, vesicles or channels, or even LNPs with diverse ionizable lipid compositions.^230^

Box 2 **Protocols for Building Complex Systems**

***CHARMM-GUI*:**(301) CHARMM-GUI, is a graphical interface that facilitates the simulation setup compatible with major molecular dynamics packages such as GROMACS,^263^ AMBER,^281^ OpenMM,^189^ and NAMD.^265^ Particularly useful for lipid-based simulations, this tool supports an extensive library of CHARMM36 lipids including bacterial and yeast-specific lipids or even multicomponent assemblies.^302−304^ Further, it is capable of preparing simulation setup with AMBER Lipid21 and MARTINI FF. This web interface^303,304^ allows the visualization of the entire system setup process and, providing equilibration and production simulation inputs, making it highly accessible for beginners.

***INSANE*:**(216) The INSANE method, short for INSert membrANE, is a command-line tool designed for constructing membrane-containing systems. Utilizing preset CG lipid templates, it efficiently builds membranes and allows users to generate simple lipid types on-the-fly. If desired, after construction and equilibration, the generated models can be converted to an atomistic membrane model.^305−307^ Hence, besides being an instrument for building CG membranes, this method offers an efficient approach for generating equilibrated atomistic models, particularly beneficial for multicomponent membranes.

***TS2CG*:**(308) TS2CG was designed to build CG membrane models by backmapping dynamically triangulated structures into molecular models. It can incorporate experimentally obtained membrane shapes and compositions to generate the initial structures. The method’s versatility allows for the generation of diverse structures, such as LNPs outer membranes and tubular lipid compartments that can be assembled into inverted hexagonal phase structures. This wide-ranging capability showcases its utility in assembling diverse membrane architectures for lipid-mediated delivery systems.

***Polyply*:**(255) Polyply is a Python suite designed to streamline the generation of input files and system coordinates for simulating (bio)macromolecules, including synthetic polymers and polysaccharides. Users can generate input files from specified building blocks or polymers available in the library. Coordinates are generated via a multiscale random-walk protocol capable of producing condensed phase systems or more heterogeneous setups such as aqueous two-phase systems. For the specific case of lipid-mediated delivery systems, Polyply can be used to generate RNA/DNA cargo molecules or the PEG chains of LNP’s PEGylated lipids.

***Packmol*:**(309) Packmol is a tool that packs molecules into predefined regions of space. It is useful for the creation of ordered systems such as lamellar, spherical, and tubular lipid layers. Users can specify the coordinates, quantities, and spatial constraints of each molecule such as lipids, drugs, and other components within a box. Packmol’s ability to handle various spatial constraints makes it valuable for constructing diverse molecular systems with precise control over their organization and structure, such as assembling tubular lipid compartments into an inverted hexagonal phase or creating an oil-like core in an LNP.

By leveraging these published tools along with established protocols, researchers can effectively construct and study the behavior of lipid-mediated delivery systems at the molecular level. This enables detailed investigations into drug distribution, carrier morphology, and interactions with biological membranes, ultimately contributing to the development of more effective drug delivery strategies.

#### 2.1.5 Enhanced Sampling Methods

AA MD simulations are a powerful tool for studying molecular behavior at the atomic level during nanoseconds to microseconds, offering detailed insights into molecular structures, dynamics, and interactions.^86^ However, classical MD simulations may struggle to capture rare events or long lasting transitions due to limited simulation time, sampling only a small region of the conformational space around the starting structure. The same applies to CG approaches but on larger time scales beyond millisecond. In complex systems, such as lipid-mediated API delivery, crucial events like API release or membrane fusion may occur infrequently,^310−313^ making them difficult to observe with classical MD alone.^314,315^ Enhanced sampling methods address this by biasing simulations to explore rare events or transitions, primarily through collective-variable-based (CV-based) or temperature-based (T-based) approaches. While this article briefly outlines their principles, limitations and predictive abilities, recent reviews provide deeper insights.^316,317^

Box 3 **Collective Variable-Based Methods**

Enhanced sampling methods based on collective variables (CVs) guide MD simulations toward specific regions of the configurational space and the transition paths between them. CVs simplify the system’s structural complexity into low-dimensional spaces that capture rare events or transitions. A CV is selected to reflect the phenomenon of interest and can range from simple parameter, like distance between two defined positions (e.g., distance of a molecule from the center of mass of a lipid membrane in membrane permeability studies),^318^ through collective descriptors like Root Mean Squared Deviation (RMSD) or eRMSD^319^ capturing global conformational changes up to complex variables, such as pathCV or property map CV.^320,321^ In some biological cases, like ligand channeling to an enzyme active site,^322^ multiple CVs are necessary. However, these methods are only effective when the relevant slow-motion degrees of freedom are captured by one or two CVs. Users must define CVs that represent the key degrees of freedom for their process,^323^ which are biased by potentials or restraints. Other degrees of freedom remain unbiased, so if an important one is omitted, the simulation may converge as slowly as standard unbiased MD simulation. Thus, CV-based methods depend on prior knowledge of the process to choose an appropriate CV path.

CV-based methods are divided into two main categories: i) those requiring multiple simulations along the chosen CV, such as z-constraint^324^ or umbrella sampling^325^ with weighted histogram analysis method,^326^ which generate a potential of mean force (PMF) describing thermodynamic preferences rather than simulating dynamic processes directly; ii) those that bias a single simulation, like adaptive biasing force^327^ or metadynamics,^328,329^ which gradually adjust a biasing potential based on already visited structures, thereby flattening the free energy profile in CV space and enhancing sampling along this direction. Both approaches provide a PMF, but if the CV does not adequately describe the process of interest, it can lead to biased results, making it necessary to reevaluate the chosen CV and check the PMF convergence.^82^

The CV-based methods are commonly used to evaluate drug-membrane interactions and predict the affinity or permeability of lipid membranes, liposome encapsulation efficiency, etc.^300,330−332^ Here, CV choice is typically straightforward, such as the drug’s distance from the lipid membrane center. Sampling may be enhanced by involving other CVs, like molecular orientation.^318^ A similar setup can evaluate the orientation of the molecules in membranes, flip-flop energy cost,^333^ or lipid affinities.^334^ In general, as long as a CV describing the process is defined, it can be biased and studied, offering many applications for lipid-mediated delivery systems. A properly chosen CV can describe and bias lipid phase transition, API encapsulation or release, fusion process^230,335^ by e.g. defining stalk formation by specified CV,^230^ etc. The resulting PMFs can quantify differences between similar systems, such as similar lipid mixtures, APIs etc. In addition to observation of the molecular mechanism of individual phenomena, such studies can also quantify their energetic cost.

Box 4 **Temperature-Based Methods**

Temperature-based enhanced sampling methods use temperature variations (or the effective temperature via potential energy scaling) under constant volume conditions to accelerate MD simulations and enhance the sampling of configurational space. By rising the system’s temperature, these methods increase the kinetic energy of the molecules, allowing them to overcome local energy barriers more readily and explore multiple regions of the configurational space. Typical examples include replica exchange molecular dynamics (T-REMD)^336^ and simulated annealing.^337^ Beyond temperature scaling, a significant subset of methods involves scaling the potential energy, which is equivalent to increasing the temperature per Boltzmann distribution. Since energy is additive, adjusting the effective temperature through potential energy scaling, applied either system-wide or selectively, offers flexibility. These are referred to as Hamiltonian-based replica exchange molecular dynamics (H-REMD),^338,339^ including methods like replica exchange solute tempering (REST2).^340^ Another group of T-based methods accelerates sampling by flooding the potential energy minima below a specified energy cutoff.^341−344^ Unlike H-REMD methods, which scale energy multiplicatively, these methods introduce an additional energy term proportional to the energy gap between unbiased potential energy and the energy cutoff value, bridging T-based and CV-based methods.

The primary limitation of temperature-based methods is that elevated temperatures (or effective temperatures from potential energy functions) help overcome enthalpy barriers but not entropy barriers. While T-based methods are highly effective for accelerating enthalpy-driven processes without prior structural knowledge, CV-based methods remain the only option for sampling of entropy-driven processes. Additionally, T-based methods may struggle to accurately capture system behavior at elevated temperatures, especially when systems are sensitive to temperature changes, such as undesired lipid phase transitions, complicating simulation convergence. Moreover, these methods require careful tuning of parameters, such as the temperature ladder in T-REMD or biasing potentials in REST2, to balance sampling efficiency without introducing artifacts. Finally, T-based methods can be computationally expensive, particularly for large biomolecular systems or when extensive configurational sampling is required.

Temperature-based enhanced sampling methods are widely used in computational biophysics and chemistry. They have been applied to study various biomolecular systems and processes, including protein folding,^345,346^ sampling dynamic protein conformation,^347^ protein–ligand docking,^348^ protein–protein recognition,^349^ description of dynamic and folding of nucleic acids,^350^ and protein structural ensemble generation.^351,352^ However, T-based methods are rarely used for lipid-based systems, except in cases like lipid mixing via REMD^353,354^ or REST^355^ and LNP assembly via simulated annealing.^297^ These methods hold potential for studying complex lipid structures such as LNP stability and lipid organization around various APIs.

##### 2.1.5.1 Future of Enhanced Sampling Methods

Both CV- and T-based methods hold great potential for simulating rare events but require experienced users to manage their limitations, as the result can be easily misinterpreted. CV-based methods with inadequate CV can result in slow, incomplete sampling. T-based methods are not suitable for entropically driven processes and must be used carefully with systems experiencing phase transitions at high temperatures. H-REMD methods offer flexibility by scaling part of the Hamiltonian to accelerate sampling without triggering an unintended phase. Enhanced sampling techniques, while useful, rely heavily on the employed FF. While enhanced sampling extends conformational sampling and improves convergence of the simulations, the FF ultimately determines accuracy; thus, using these methods along with an inaccurate FF only amplifies the likelihood of FF-related artifacts. From a certain point of view, both types of enhanced sampling methods are complementary, favoring CV-based methods for studies of processes along a well-defined pathway and T-based methods for increasing the configurational space sampled without prior knowledge of a desired final state. The future lies in the synergy between these two types of enhanced sampling techniques.^356,357^ Used together, these approaches can help to address complex biomolecular problems more effectively. One example of a hybrid method involves integrating REST2 or REMD with external biasing potential methods. This combination enables enhanced sampling of rare events and transitions while also biasing the simulation toward specific regions of interest.^358^

Alternatively, enhanced sampling techniques can be combined with other computational approaches (such as ML described in the following section) as a powerful approach for studying complex biomolecular systems like LNPs.^359,360^ One effective strategy is to leverage data-driven signals to boost molecular sampling and overcome the computational resource constraints of MD simulations. For instance, ML methods can be employed to predict system properties such as interaction forces and potential energy surfaces and these predictions can then be incorporated into MD sampling, allowing for more efficient sampling of the conformational space and guiding the sampling process toward regions of interest, such as reaction coordinates or structures formed by molecular assemblies.^361−363^ This approach has proven to yield valuable insights in various molecular systems.^362,363^ A notable pioneering example is the combination of predicted coevolutionary signals from genetic sequences with MD sampling to drive the characterization of protein conformational changes and functional folds, understand fold mechanisms and large complex assemblies.^364−370^

Moreover, MD sampling can serve as input features for ML models.^363,371,372^ MD simulations generate trajectories that capture the time evolution of atomic positions and velocities, and data-driven models can extract meaningful information from these trajectories. For instance, machine learning algorithms can be trained on MD data to predict potential energy surfaces, free energy landscapes, or properties like solubility and binding affinity.^371,372^ One particularly promising approach is the use of physically informed neural networks (PINNs).^373^ PINNs incorporate physical laws and constraints into the neural network architecture, ensuring that the model predictions are consistent with underlying physical principles and constraints derived for example from MD simulations.^373,374^ This can enhance the accuracy and generalizability of ML models for investigating biomolecular systems.

### 2.2 Data-Driven Statistical Methods

#### 2.2.1 History from QSAR to Deep and Machine Learning

The physical, chemical, and biological properties of molecules are intrinsically linked to their chemical structure. Establishing quantitative correlations between these properties and chemical structure is critical for evaluating, understanding, and utilizing new chemical entities from drug discovery to materials science. Quantitative structure–activity relationships (QSAR) and quantitative structure–property relationships (QSPR) provide a framework to link molecular activities to structural features, enabling systematic exploration of chemical space.^375^

The concept of QSAR originated in the mid-20th century when scientists first examined the relationship between the chemical structure and biological activity. Foundational models, such as those proposed by Hansch and Fujita, utilized linear regression to correlate physicochemical properties (such as lipophilicity or electronic characteristics) with biological outcomes. These foundational efforts paved the way for advanced QSAR models that quantitatively predict how structural features influence molecular activity.^375,376^

Recent years have seen the evolution of QSAR methodologies, driven by advances in cheminformatics, structural biology, and ML. Leveraged by the era of big data, ML has emerged as a transformative gradually improving tool, capable of automatically recognizing complex and hidden patterns from large data sets, rather than relying on directly coded instructions. Most ML implementations rely on supervised learning, which uses labeled data sets to train predictive models and iteratively adjusts their underlying parameters to match the observed data patterns and labels (a process known as ML training step). These trained models are evaluated for their ability to predict labels of previously unseen data and can forecast outcomes for data with unknown labels. They can also be used to investigate identified patterns and aid in decision-making. Consequently, as a method that “learns from the past”, supervised ML methods are limited by the amount and quality of available data, as well as the diversity of system states represented within data sets. More details on supervised and other ML learning methods can be found elsewhere.^377−379^

The expansion of computational power has facilitated the adoption of nonlinear ML algorithms including support vector machines, random forests, and neural networks. These nonlinear ML methods operate by fitting complex nonlinear functions to the data. This process is analogous to nonlinear least-squares fit, where a specific nonlinear function is chosen and optimized to minimize the error between the model’s predictions and the actual data. However, unlike traditional nonlinear least-squares fits, ML methods do not require the explicit specification of a functional form as proposed solution to describe the system. Instead, ML learns the optimal function directly from the data.^355,356^

Large chemical databases and high-throughput screening data fueled further advancements.^380^ The integration of diverse data sources, including protein–ligand interactions, protein structures, and omics data, into multidimensional QSAR models represents a current trend yielding to more accurate predictions and deeper insights into molecular mechanisms. Efforts to improve QSAR model interpretability and transparency continue, ensuring that QSAR approaches remain integral to drug discovery, environmental risk assessment, and materials science, bridging the gap between chemical composition and biological function.^381^ In the field of lipid-based delivery, especially in LNP design for mRNA delivery, the evolution of QSAR with ML offers a powerful approach to navigate the complex design space. It can provide invaluable insights into the functional contributions of individual LNPs, enabling efficient identification of promising LNP candidates for further exploration.

#### 2.2.2 Machine Learning for LNP Design

The rational design of LNPs for mRNA delivery presents a complex challenge, as multiple components must work in synergy not only to promote functionally assembled LNP structures able to carry mRNA payloads, but also to dynamically respond to their environment; triggering specific molecular mechanisms such as LNP disruption after cellular uptake, allowing endosomal escape and mRNA transfection.^15^ Considering the number of variables to explore, ML emerges as a method with immense potential to accelerate the design of novel and optimized LNPs. Elucidate the contributions of individual component, ML can provide insights into LNP mechanism,^382^ delivery efficacy,^383^ and a growing body of features captured by ML models across data sets.^384^ Examples of key LNP properties that can be fine-tuned with an ML-aided design include transfection efficiency, tissue targeting, vaccine thermostability, and reactogenicity.

Though LNPs have been extensively studied as delivery systems for genetic material for decades,^15,385^ the first attempts to leverage ML for rational LNP designs were only reported very recently, boosted by the development of COVID-19 mRNA vaccines.^15^ LNPs typically consist of four primary lipid components: ionizable lipids, helper lipids, PEGylated lipids, and cholesterol. Among these, ionizable (or cationic) lipids are the primary focus, since they are regarded as a main component of LNP with a major impact on both mRNA stabilization and endosomal escape and thus on the LNP’s overall transfection efficiency. ML can investigate the role of each structural component, optimizing their combinations to balance efficacy, biodegradability, stability, and other developability criteria. Integrated into an iterative experimentation pipeline, ML methodologies have demonstrated the capability to direct the efforts toward highly developable structures, significantly reducing costs.^386,387^ Future investigations exploring novel LNP compositions beyond the currently used four major components may drastically change LNP applications.

*In silico* approaches have adopted graph neural networks to model spatially aware molecular representations,^388^ not only in small molecule design^389^ but also in LNPs payload, such as RNA.^390^ Neural small-molecule fingerprints, generated through large-scale, weakly supervised deep learning on structural databases, have shown performance on par with expert-derived fingerprints in various domains beyond the LNPs.^391^ Property prediction models, integral to small-molecule chemical design, are increasingly being developed for LNP applications,^392^ as well as the application of transfer learning, where generalizable concepts learned from small-molecule models are adapted to the LNP design.^387^

One of the first approaches using ML to design new LNP components was performed by Ouyang et al. in 2022.^392^ In this study, a collection of 325 measurements of IgG titer levels (a surrogate of antibody production by injected mRNA, as a measure of transfection efficiency) for distinct LNP formulations was prepared, and a supervised regression model was developed to predict IgG levels based on the LNP formulation. The LNP molecular components were encoded as part of model input using extended connectivity fingerprints representation,^393^ and a trained ML model employing LightGBM^394^ algorithm based on gradient boosting presented an average determination coefficient (*R*^2^) of 0.87 for a 10-fold cross validation.^392^ In a similar study employing a larger curated data set of 622 LNPs from previous studies, Sun et al. implemented and trained a Multi-Layer Perceptron algorithm to predict transfection efficiency of new LNPs, achieving a classification accuracy of 98% on the test set.^384^ Another approach for LNP optimization was reported by Jeong et al.^395^ This study focused on using ML to predict and optimize physicochemical properties of LNPs such as particle size, polydispersity index, zeta potential, and encapsulation efficiency from input parameters including LNP component types, ratios, and also flow ratios during lipid mixing. Overall, the final multitarget regression ML model based on neural networks presented R^2^ predictive coefficient ranging from 0.72 to 0.99 depending on the target.^395^ Finally, a recent work from Li et al. proposes an end-to-end open-source pipeline solution to assist the design of novel ionizable lipids for mRNA delivery.^387^ This platform, called AGILE (AI-Guided Ionizable Lipid Engineering) provides not only an implementation for training deep learning models to screen new ionizable lipids but also a pipeline based on combinatorial chemistry to generate large libraries of new lipid scaffolds for lipids discovery. In a real-case implementation, the authors demonstrated the applicability of the AGILE platform by identifying 15 ionizable lipids candidates from a screen of 10,000 potential candidates, validating optimized transfection efficiency through *in vitro* and *in vivo* experiments.^387^

In summary, the rational development of LNPs for mRNA delivery involves a complex design space, where multiple components must work synergistically to carry mRNA payloads and dynamically response to environmental triggers.^15^ Consequently, the study of LNPs requires the exploration of numerous factors influencing the observed efficacy and, simultaneously, the safety of vaccines. In this context, ML emerges as a method with immense potential to accelerate the screening and design of novel and optimized LNPs and to help elucidate the contributions of each component to observed outcomes.

## 3 Overview of Computational Modeling of Lipid-Mediated Delivery Systems

The following section outlines the current state-of-the-art in liposomal and LNP simulations, highlighting their application potential to complement experimental approaches (Box 5). Specific lipid-based structures, such as lipid nanodroplets,^396^ are not covered here.

Box 5 **Computational Insight into Experimental Data**

With the exponential growth of computational resources, a wide array of computational tools, fine-tuned parameters, and advancements in fields such as ML have positioned computational techniques as a powerful complement to experimental methods. This is particularly valuable when experiments face limitations or when studying inaccessible phenomena. Computational techniques offer insights into the structure of complex liposomes^469^ or PEGylated liposomes, providing a detailed understanding of their molecular architecture and dynamics at atomistic resolution (Figure 4B).^454^ Especially for LNPs, where the resolution of cryo-EM cannot fully describe the internal molecular organization, MD simulations can enhance experimental data by revealing LNP organization from large-scale spacing in the H_II_ phase to a detailed insight into the distribution of contacts between individual molecules.^297^

The role of computational insights becomes even more significant in highly dynamic processes such as the self-assembly of liposomes and other lipid-based carriers. Understanding these formation processes can enable the design and control of effective drug delivery systems (Figure 4C).^414^ Dynamic molecular insight into the structure of LNCs can clarify their thermal stability, hydration evolution, responses to specific triggers, and lipid exchange ratios during LNC-membrane fusion.^334^ In the near future, it may be possible to identify the species responsible for the fusion of specific lipid mixtures or to rationalize the cargo release process.

Computational techniques have rapidly evolved into essential tools for advancing lipid-mediated drug delivery, offering perspectives that complement and enhance traditional experimental approaches. They enable prediction of drug release from lipid carriers under various conditions, such as temperature, pressure, and precisely tailored lipid composition, supporting the design of formulations for controlled release (Figure 4A).^422^ By enabling the precise customization of individual components, these computational techniques provide predictive insights into LNC formulation, stability, encapsulation, and cargo release. This approach has the potential to advance our understanding of lipid–mediated drug delivery and streamline the development of effective therapeutic strategies.

### 3.1 Liposomes

Given the relatively large size of liposomes (ranging from tens of nanometers up to micrometers in diameter),^23^ a feasible computational approach to address liposomal-based drug delivery is to downscale the system to simplified models of lipid bilayers at atomistic resolution. This allows for the prediction of drug permeabilities and partitioning.^397−402^ The bilayer approach can also be adapted to mimic curvature effects within certain limits.^403^ The downscaling to lipid bilayers provides precise yet computationally achievable tools for predictions, which can be extrapolated back to liposomes to understand the efficient encapsulation of compounds and subsequent cargo release. While the use of simplified lipid bilayer models has been reviewed elsewhere^64,404^ and several comprehensive reviews on liposomes have been published recently,^64,86,401,405^ this section focuses on the potential of liposome simulations as discrete particles on more realistic spatial scales.

Liposomes are often studied using CG representation due to their ability to efficiently capture the essential dynamics of complex, large biomolecular systems over long time scales at a reasonable computational cost.^406^ Only a minority of studies use AA^407−410^ or UA^411^ representation to model liposomes. Liposomes composed of phospholipids and/or cholesterol have been prepared through various methods, including the aggregation of randomly distributed lipids,^196,231,411−420^ the spontaneous formation of liposomes from an initial bilayer^415,421,422^ or bicelle,^412,423,424^ or the prebuilding of structures using various tools.^303,408,409,425,426^ These simulations focused on the role of individual lipids in liposome structure and formation, demonstrating, for example, that the lipids with cylindrical geometry tend to form spherical liposomes.^414,427,428^ The concentration of unsaturated lipids can increase the probability of spontaneous liposome formation from a bicelle,^424^ and mixture of lipids with different saturation levels can lead to lipid domain separation.^429^ Lipids with a small headgroup, such as PE^416,430^ or cholesterol,^419,431^ preferentially occupy the inner liposomal leaflet, and the lipid domains formed on a flat bilayer can induce lipid curvature, sometimes even leading to the spontaneous spherical liposome formation.^421^ The concentration of PEGylated lipids also influences aggregate size,^214^ shifting from liposomes to PEGylated micelles as PEGylation levels increase. Overall, lipid composition affects the size, shape, and general structure of the resulting liposome.

Simulations have been used to assess liposomal stability under specific conditions and its response to disruption events. These studies show that the increased liposomal membrane curvature promotes a fluid phase^418,432^ and reduces the energetic cost of pore formation.^433^ Raising the temperature in PC/PE mixture accelerates liposome formation, thus lowering the computational cost,^423^ while in liposomes containing elastin-like polypeptides, higher temperatures cause random coil formation, which disrupts the liposomal stability.^419^ Increased temperature or ethanol content has also been shown to reduce liposomal stability,^434^ whereas incorporating octanoylated hyaluronic acid increases it.^435^ In contrast, adding a photosensitizer can induce a rich hydrogen bonding network, thereby increasing liposome rigidity.^409^ Liposomal rigidity may also affect response to ultrasound, leading to liposomal leaflet detachment in liposome containing CHOL or pure DOPC liposome, and pore formation in pure DPPC and POPC liposome.^408^ simulations further allow precise manipulation of LNC composition, which has been used to monitor the effect of liposomal core’s hydration level on liposomal rupture^436^ or liposomal shape,^437^ a phenomenon that can be experimentally induced by osmotic pressure. Overall, these simulations provided insight into molecular organization and helped explain experimental observations.

In addition to assessing the stability of liposomal formulations, CG simulations offer detailed insights into the encapsulation and distribution of APIs or even interaction with nanomaterials.^438,439^ These simulations can unravel the effect of the number of loaded compounds on the size of the liposome and the effect of the protonation states and polarity of drugs on their partitioning between the lipidic and aqueous phases of the liposome. MD simulations have confirmed the experimentally observed size increase of the liposomal carrier with an increasing amount of loaded cargo.^440^ Furthermore, they have elucidated the relationship between carrier-cargo properties, such as cargo polarity, showing the preferential distribution of less polar compounds into hydrophobic tails region and more polar compounds into the hydrophilic area below the lipid headgroup region or directly into the aqueous core of liposomes.^330^ Similarly, studies focused on cargo protonation states indicated the predominant distribution of neutral compounds into the hydrophobic regions of the vesicle between the internal and external vesicle monolayers, whereas the protonated compounds preferred the water phase.^441^ PMF calculated on the whole liposomes reveal a slight preference of large molecules for the outer liposomal leaflet.^442^ These studies have enhanced our understanding of cargo encapsulation mechanisms, consequently aiding in the refinement of delivery schemes.

MD simulations have furthered our understanding of the mechanisms and roles of individual components in the liposomal fusion process, which is crucial for cargo delivery.^410^ CG simulations revealed at a molecular level that fusion was initiated by extending lipids at the lipid–lipid contact point, leading to stalk formation.^443−447^ Elastic^448^ or smaller liposomes fused faster,^443,449,450^ a phenomenon that can be quantified^451^ by calculating the height of the energy barrier for stalk formation, which decreased with increasing lipid curvature.^452^ The presence of lipids with negative spontaneous curvature, such as PE, also reduced the energy barrier for stalk formation.^452^ PE lipids were found to assemble around the stalk or in the internal monolayer of the liposome.^274^ The liposomal composition also affected the propensity to fuse. For instance, while liposomes composed of cylindrical PC lipids repelled each other, PE-containing liposomes fused spontaneously.^274^ Similarly, cholesterol was shown to induce liposomal-membrane fusion.^453^ However, the most efficient fusion occurred when either the liposome or lipid membrane was in a cholesterol-rich state. If both or neither were cholesterol-rich, fusion was limited, and endocytosis may be preferred.^453^ Further, large scale MD simulations were used to rationalize the role of membrane surface tension and ligands on PEGylated liposomes on the process of liposome wrapping.^454^ Insights into the fusion mechanism can bring us closer to developing highly efficient liposomal formulations.

### 3.2 Lipid Nanoparticles and Other Soft LNCs

Rapid advances in nucleic acid delivery by LNPs are still hindered by their limited delivery efficiency, highlighting the critical need for the rational design of LNP formulations. Despite the known composition of LNPs, their spatial organization remains mostly unknown. Computational approaches offer insights into both of these aspects, complementing experimental data by providing a molecular picture of the LNPs and simulating their assembly and action. As recent reviews have covered individual LNP simulations in detail,^455^ our focus here is on their general outcomes, with Table 3 providing a summary of LNP studies categorized by model complexity and IL type.

**Table 3:** Categorization of LNP Simulation Literature by Size of the System

Simulation Category	Cationic/Ionizable Lipids studied	ref
Simulations of LNP Bilayer Systems	KC2	Ramezanpour,^40^ 2019
	MC3	Ermilova,^99^ 2020
	KC2 and MC3	Park,^110^ 2021
	SM-102 and ALC-0315	Paloncýová,^456^ 2021
	ALC-0315	Trollmann,^298^ 2022
	SM-102, ALC-0315, and MC3	Ermilova,^100^ 2023
	Lipid-5	Dehghani-Ghahnaviyeh,^410^ 2023
	Large family of literature known ILs	Kjølbye,^230^ 2024
		
Simulations of LNP Core Organization	General CL	Farago,^460^ 2006
	DMTAP	Khalid,^461^ 2008
	DOTAP	Corsi,^462^ 2010
	KC2	Leung,^463^ 2012
	SM-102 and ALC-0315	Paloncýová,^456^ 2021
	KC2	Ramezanpour,^458^ 2022
	Lipid 1, 29, 34, 36, 39, and 40	Cornebise,^175^ 2022
	MC3	Kjølbye,^230^ 2024
	LP01	Garaizar,^466^ 2024
		
Simulations of the Whole LNP Systems	DOTAP	Bruininks,^459^ 2020
	KC2	Leung,^463^ 2012
	ALC-0315	Trollmann,^298^ 2022
	ALC-0315	Grzetic,^210^ 2024
	ALC-0315	Paloncýová,^297^ 2023
	MC3, Lipid 2, and Lipid 10	Kjølbye,^230^ 2024

As with other lipid-based systems, simple bilayer models are often used to study LNP systems. In bilayers mimicking the lipid composition of LNPs, the headgroups of cationic ILs remained at the bilayer-water interface, with lipid tails incorporated among the tails of the other bilayer lipids. This pattern holds for the first generation ILs like MC3^32,110^ and KC2,^40,110^ as well as for the ILs found in the Pfizer and BioNTech (ALC-0315^298,456^) and Moderna (SM-102^456,457^) COVID-19 vaccines. In their neutral form, ILs showed a high tendency to accumulate in an oil phase between leaflets,^40,110,298,457^ consistent with experiments showing that the core of KC2-LNP was partly composed of neutral KC2 lipids.^75^ However, the phase separation of ILs was observed to be concentration-dependent and did not occur at low IL concentration.^99,100,457^ In systems with PEGylated lipids, the PEG-chains interacted with IL headgroups, due to the attractive interactions between their head groups and the PEG oxygen.^110^

**Figure 3:**
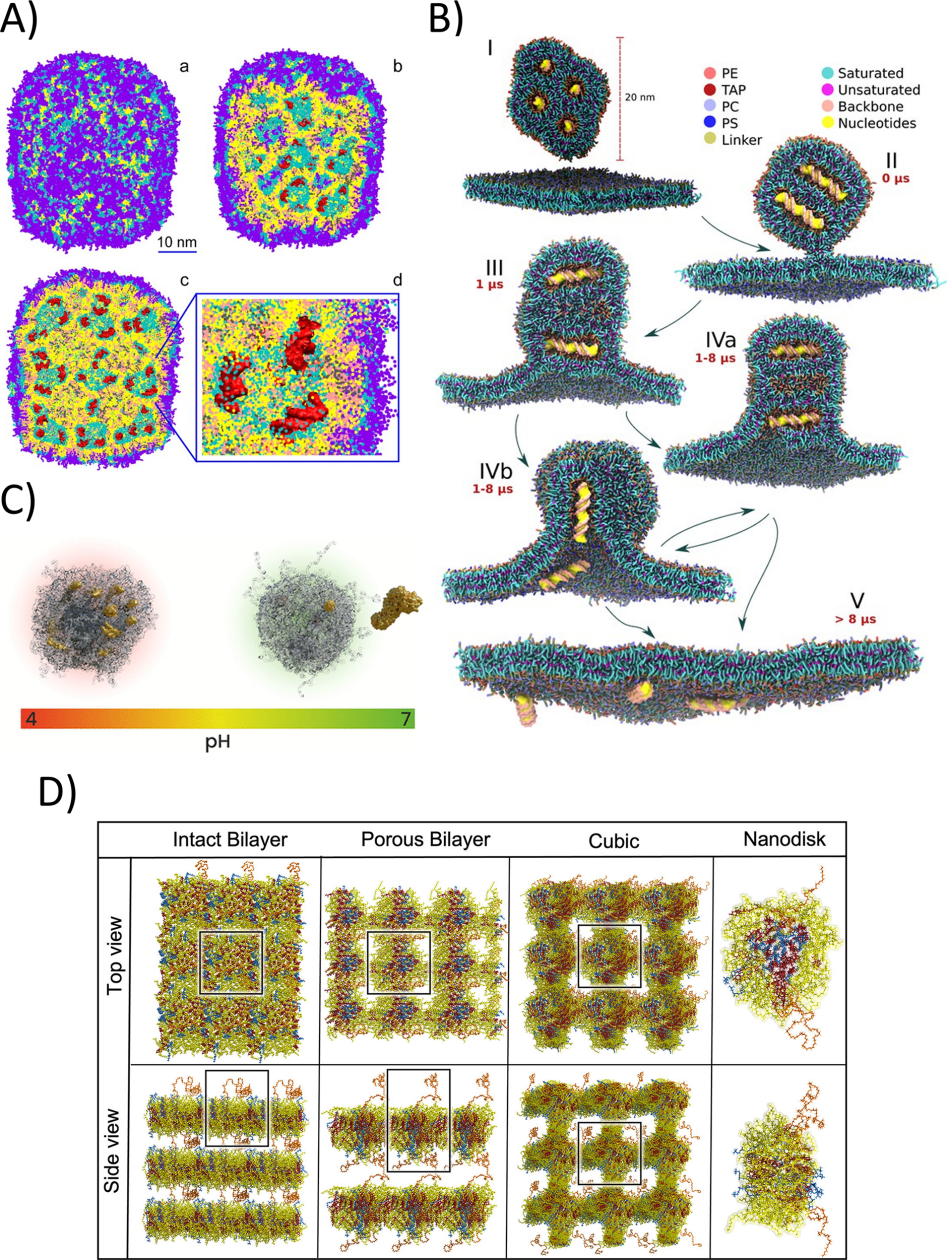
(A) LNP assembled by replicating a building block generated by self-assembly of a mixture of KC2, DSPC, cholesterol, and nucleic acids (shown in the purple square). The resulting structure was coated with a PEGylated lipid layer. Reproduced from ref (463). Copyright 2012 American Chemical Society. (B) Schematic view of the lipoplex-membrane transfection pathways from unadhered lipoplex with four dsDNAs above the endosomal bilayer (I) to the release (IVa) or ejection (IVb) of DNA (V). Reproduced from ref (459). Available under CC-BY 4.0. Copyright 2020 Bruininks et al., published by eLife Sciences Publications Ltd. (C) Protonated (on the left) and deprotonated (on the right side) LNPs showing the tendency of LNPs to exclude RNA at neutral pH. Reproduced from ref^297^. Copyright 2023 American Chemical Society. (D) Possible lipid organizations resulting from self-assembly of systems containing ILs are shown in yellow, cholesterol in red, DSPC is in blue, and PEGylated lipids in orange. Reproduced from ref (456). Copyright 2021 American Chemical Society.

MD simulations of IL-containing bilayers seem to be stabilized by periodic boundary conditions. In self-assembly simulations, when mixed with cholesterol and DSPC ILs like ALC-0315 and SM-102 preferred regions of high curvature of nonlamellar phases, like the inverted hexagonal phase (H_II_, Figure 3D).^456^ H_II_ consisted of hexagonally packed water channels surrounded by lipids, with the ILs polar heads close to the water channel and the hydrophobic tails extending outward. The stabilization of this H_II_ phase was investigated atomistically in mixtures containing protonated KC2, cholesterol, and DSPS, with varying molar ratios of each component.^458^ It was found that cholesterol tended to colocalize with the saturated DSPS lipid, enhancing its tail order and stabilizing the H_II_. In contrast, KC2, with its shorter polyunsaturated tails, weakly localized in the space between two adjacent tubes. The authors proposed that the stabilization of the H_II_ phase was facilitated by cholesterol and the lipid chain saturation, which was consistent with previous descriptions.^459^

The H_II_ phase is also stabilized by the presence of nucleic acids. Pioneering self-assembly simulations showed that, at high concentrations, cationic lipids (CLs) formed pores in the membrane, allowing DNA translocation.^460^ Subsequent studies using more detailed CG models^211^ revealed that dsDNA aligned at the water–lipid headgroup interface,^461^ inducing a transition from a lamellar to a H_II_ phase.^459,462^ An identical behavior was seen in self-assembling LNPs containing KC2,^463^ ALC-0315,^298,456^ or SM-102^456^ with RNA fragments, DSPC, cholesterol, and PEGylated lipids. Under low hydration levels, protonated KC2 molecules organized into inverted micelles with siRNA, closely resembling a disordered hexagonal phase (Figure 3A).^463^ In smaller systems, nucleic acids tended to adhere to the heads of charged ILs, remaining on the lipid surface.^456^ Headgroup-modified Moderna ILs improved transfection and exhibited increased interactions through π-stacking between the modified planar IL headgroups and adjacent nucleobases.^175^

These small-scale models of lipid mixtures with nucleic acids laid the foundation for constructing a complete LNP.^298,463^ A pioneering CG study^463^ built siRNA containing LNP via self-assembly, which was then multiplied into a roughly spherical particle and coated with a lipid monolayer that included PEGylated lipids. Similarly, Trollmann et al.^298^ multiplied an AA structure of an oil phase containing neutral ALC-0315 and cholesterol, enforced into a spherical shape and covered with a mixture of protonated IL, DSPC, cholesterol and PEGylated lipid. To gain insight into the LNP organization of the Pfizer BioNTech COVID-19 vaccine, self-assembly simulations were conducted to replicate the LNP behavior during synthesis.^297^ In these CG simulations, conical ILs assembled around siRNA to form a H_II_ phase with multiple water droplets, with RNA fragments at the core, resulting in a ring-like structure consistent with onion-like model of LNP.^75^

An important tool to capture pH-induced changes is manipulating IL protonation through MD simulations. LNPs are initially prepared in an acidic environment, which promotes IL-nucleic acid interactions; however, during storage and LNP administration, LNPs exist in a neutral environment. Upon reaching the target tissue, they encounter a pH shift, from early endosomes (pH 5.5–6.5), to late endosomes (pH 5.0–5.5), and finally to lysosomes (pH 4.5–5.5).^464^

Protonated ILs were described to preserve a hydrated H_II_ phase (Figure 3C, left side), however, upon deprotonation of the ILs, rapid structural changes occurred in the LNPs, causing the release of most of the encapsulated water and RNA (Figure 3C, right side).^297^ Upon reprotonation, mimicking endosomal maturation, the LNPs quickly swelled and rehydrated,^297^ potentially aiding the endosomal escape. Simulations of the Pfizer/BioNTech vaccine LNP also demonstrated that protonation levels of ILs significantly influenced structural stability.^210^ High protonation levels (over 50%) resulted in minimal structural changes, from the fully protonated LNP organization, while lower levels (40% to 0%) lead to “bleb” formation,^210^ aligning with experimental observations in RNA-loaded LNPs.^38,465^

After endosomal uptake, LNPs must fuse with the endosomal membrane to release their cargo. Similarly, when IL-containing nanodroplets come into contact, they induce mixing of lipids from membrane and nanodroplet models.^334^ When simulating KC2-containing systems, the CL charge seems to facilitate adhesion to the endosomal membrane and initiate fusion.^458^ CG Martini^212^ simulations of a 20 nm LNP with dsDNA also demonstrated that upon close contact and guided formation of fusion stalk, fusion led to successful transfection of the dsDNA cargo (Figure 3B).^459^ These simulations highlighted the delicate equilibrium between structural stabilization and destabilization. Unsaturated ILs were found to stabilize the H_II_ phase within the LNP, thereby promoting successful transfection, whereas saturated ILs tended to destabilize it, diminishing transfection efficacy. Recent Martini 3 models of ILs have also demonstrated significant potential for elucidating the effects of critical parameters, such as pH and composition, on the structure and efficiency of LNPs. These were obtained both by simulating the LNP core^230,466^ and entire LNPs,^230^ offering deeper insights into their behavior and optimization.

Apart from “classical” LNPs for nucleic acid delivery, MD simulations can also explore other LNC, such as lipid-based nanodiscs, with components commonly found in LNPs^467^ or lipid nanoemulsions^468^ for drug delivery to tumors and skin. Lipid nanodiscs containing cyclic polynucleotide bound to PEGylated lipids exhibited higher penetrability to tumors compared to PEGylated liposomes and the MD simulations were used to evaluate the process of permeation of the nanodisc and liposome through a rigid pore.^467^ Lipid nanodiscs were able to adjust their shape and permeate through the pore better than liposomes. For skin delivery of vitamins A and E, a lipid nanoemulsion droplet was prepared by self-assembly simulations and the onion-like internal organization of the individual components was revealed.^468^ These kinds of simulations of complex LNCs open the path to further *in-silico* design of targeted drug delivery.

**Figure 4:**
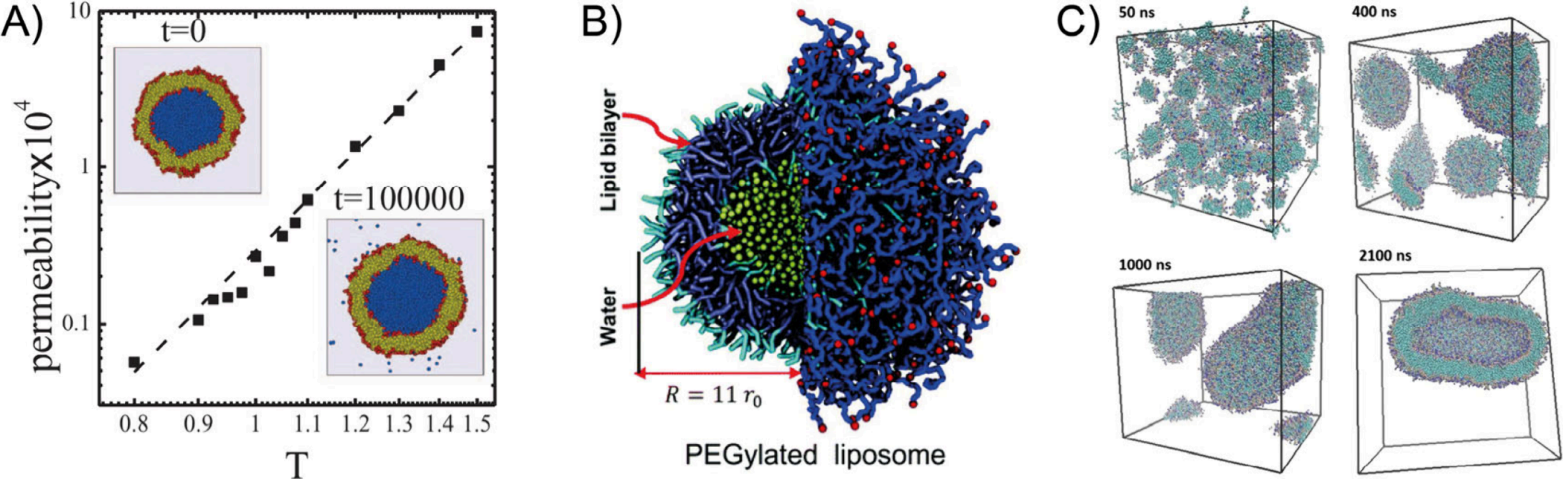
Examples of computationally derived data that are directly related to the experimental procedures. (A) The effect of temperature on liposomal cargo release. Reproduced with permission from ref (422). Copyright 2012, AIP Publishing. (B) Detailed molecular representations of PEGylated liposomes showing their internal structure and the radii of the prepared liposomes. Adapted with permission from ref (454). Copyright 2019, Royal Society of Chemistry. (C) The vesicular formation underlying self-assembly. Reproduced from ref (414). Available under CC-BY 4.0. Copyright 2022, Parchekani et al., published by Springer Nature.

## 4 Summary and Outlook

In drug delivery, extensive research has focused on unveiling the best approaches to ensure that APIs accurately reach their targets and not to other locations. The encapsulation of APIs into LNCs has revolutionized this field by enabling the design of carriers with enhanced safety, efficacy, and targeted delivery. Pioneers in this field are liposomes, which with their versatile structure can accommodate both hydrophobic and hydrophilic APIs, paving the way for the successful delivery of numerous therapeutic agents. Meanwhile, LNPs have emerged as a preferred carrier for nucleic acids, highlighted by their pivotal role in mRNA-based COVID-19 vaccines. Designing LNCs involves carefully considering the ideal lipid composition, surface functionalization, and environmental triggers to achieve a controlled release. Computational methods play an indispensable role in the development of LNCs offering unique structural and mechanistic insights that so far are not amenable to experimentation. They can also be used as predictive tools for testing LNCs composition, physical-chemical properties, and mechanism of action. LNCs, however, represent challenging systems for computational methods due to their large size and structural complexity. Since data on lipid compositions and delivery efficiency of LNCs is not easily available, generating databases for these parameters would greatly benefit future machine-learning and data-driven approaches, enabling more accurate and predictive modeling and helping to understand the connection between LNCs structure and dynamics and transfection efficiency. Due to the recent progress reviewed here, one can develop a suitable model representing the key features of a system upon investigation. Classical MD simulations offer atomic-level resolution, allowing the exploration of molecular interactions within LNCs to very fine details. However, the FF and environmental conditions must be carefully addressed to ensure the reliability of the simulation results. The price paid for the fine atomistic resolution in such large systems is the available time scale limiting configurational sampling. In contrast, coarse-grained simulations can model larger systems over longer time scales, at the expense of fine structural details. In principle, both approaches can be integrated, and one can expect that multiscale modeling approaches will play an important role in simulations of large and complex molecular systems in the near future. Another promising avenue is the integration of physics-based and data-driven approaches. Data-driven methods reached maturity in providing useful predictions and rationale for experimental observations. MD simulations generate giant data sets, which can be used to train ML models and support data-mining methods to analyze complex molecular systems such as LNCs. Without a doubt, computational methods will continue to play a crucial role in the development of lipid-mediated drug delivery. In future studies, novel lipid-based materials with well characterized biophysical properties will significantly expand the toolbox for LNC design. Moreover, interdisciplinary collaborations among computational scientists, experimentalists, and clinicians will facilitate the translation of computational insights into practical applications, accelerating the development of a new generation of LNCs for personalized medicine. In conclusion, a well-established synergy between experimental and computational approaches will be essential to driving innovation in lipid-mediated drug delivery, ushering in a new era of targeted and highly efficient therapeutics. As computational techniques continue to evolve and more computational resources become available, the capacity for predictive modeling and rational design of LNCs will be substantially enhanced. This presents new opportunities to address unmet clinical needs and improve patient outcomes. Enhanced sampling methods promise to capture long-time scale phenomena, bridging the gap between simulation and experimental time scales. This advancement will be crucial for obtaining a more accurate representation of LNC behavior over time, ultimately aiding in the design of more effective and reliable drug delivery systems. As computational power increases and methodologies become more refined, the integration of these approaches will undoubtedly lead to significant breakthroughs in the field.

## Abbreviations

API: active pharmaceutical ingredient
mRNA: messenger RNA
cryo-EM: cryo-electron microscopy
LNP: lipid nanoparticle
LNC: lipid nanocarrier
siRNA: small interfering RNA
dsDNA: double stranded DNA
PEG: polyethylene glycol
IL: ionizable lipid
CL: cationic lipid
MD: molecular dynamics
FF: force field
AA: all atom
CG: coarse grained
UA: united atom
RESP: Restrained Electrostatic Potential
PC: phosphatidylcholine
PE: phosphatidylethanolamine
PG: phosphatidylglycerol
PS: phosphatidylserine
PUFA: polyunsaturated fatty acids
SM: sphingomyelin
RESP: Restrained Electrostatic Potential
CGenFF: CHARMM General Force Field
DPD: dissipative particle dynamics
SPICA: Surface Property fitting Coarse graining
DLPC: dilauroylphosphatidylcholine
DMPC: dimyristoylphosphatidylcholine
DPPC: dipalmitoylphosphatidylcholine
DOPC: dioleoylphosphatidylcholine
DSPC: distearoylphosphatidylcholine
DSPS: distearoylphosphatidylserine
POPC: palmitoyloleoylphosphatidylcholine
POPE: palmitoyloleoylphosphatidylethanolamine
PEPC: palmitoylelaidoylphosphatidylcholine
KC2: DLin-KC2-DMA
MC3: DLin-MC3-DMA
CPU: central processing unit
GPU: graphics processing unit
PME: particle mesh Ewald
HPC: high-performance computing
CV: collective variable
CV-based: collective variable-based
T-based: temperature-based
PMF: potential of mean force
T-REMD: replica exchange molecular dynamics
H-REMD: Hamiltonian-based replica exchange molecular dynamics
REST2: replica exchange solute tempering
QSAR: quantitative structure–activity relationship
QSPR: quantitative structure–property relationship
ML: machine learning
H_II_: inverted hexagonal
